# From defense to reconstruction: The hostility-meaning dual-path model of how observing others’ adversity influences distress disclosure

**DOI:** 10.1371/journal.pone.0350109

**Published:** 2026-06-08

**Authors:** Xiaoying Zhang, Tian Huang, Yuting Wang, Zhijun Hou, Jiaqi Fu

**Affiliations:** 1 School of Medicine and Nursing, Changjiang Polytechnic, Wuhan, Hubei, China; 2 China University of Geosciences (Wuhan), Wuhan, Hubei, China; 3 China University of Petroleum-Beijing at Karamay, Karamay, Xinjiang, China; 4 Xinjiang University of Technology, Hotan, Xinjiang, China; Amity University Kolkata, INDIA

## Abstract

**Objective:**

This study explored the mechanism by which observing peers’ adverse experiences influences distress disclosure of negative information through two progressive studies.

**Methods:**

Study 1constructed a moderated mediation model to analyze the relationships among stress, self-esteem, hostility, and disclosure. Study 2 employed a situational experiment to test a chain mediation model which supplemented by multi-group analysis.

**Results:**

Study 1 showed that stress directly inhibited disclosure and indirectly inhibited it via reduced self-esteem, with hostility moderating the stress–self-esteem relationship. Study 2 confirmed a full chain mediation effect under adversity perception, with significant path coefficient differences across groups: participation willingness promoted disclosure in the adversity group but inhibited it in the prosperity group.

**Limitations:**

This study did not examine the moderating effects of cultural values, nor did it control for potential confounding variables such as gender, age, social desirability, and self-selection bias.

**Conclusions:**

This research reveals the dual-path mechanism of imperfect models: they can trigger either defensive hostility or meaning reconstruction depending on situational perception, expanding the application of the Meaning Maintenance Model in the study of role model effects and informing psychological interventions.

## 1. Introduction

In a highly digitalized social environment, individuals are increasingly frequently exposed to the meticulously presented “success narratives” of others [1]. Research indicates that self-presentation on social media platforms typically exhibits a significant positive bias, with people being more inclined to share successful, happy, or idealized life snippets, while less frequently displaying negative experiences [2,3]. This selective presentation leads users to encounter a large number of beautified life “highlight reels” while browsing information, thereby forming an idealized perception of others’ life circumstances. Empirical studies have found that the more frequently individuals use social media, the more likely they are to believe that others are “happier and have better lives” than themselves, thus overestimating others’ happiness levels and underestimating their own quality of life [1]. Within this information environment, individuals are more prone to engage in upward social comparison, which involves comparing oneself to others perceived as more successful or happier [2]. Further research shows that idealized content on social media significantly strengthens this tendency towards upward comparison and may trigger a decline in self-evaluation and negative emotional experiences [4]. In this environment, people are often described as being caught in “Duck Syndrome”: appearing calm and confident on the surface, while internally enduring stress and anxiety. A substantial body of research indicates that frequent upward social comparison lowers self-esteem and increases negative emotions such as stress, anxiety, and depression [2,5].

However, within this culture of “success narratives,” individuals facing failure or stress often tend to conceal their vulnerabilities rather than actively express negative experiences [6–8]. Research indicates that people worry that negative disclosure might bring risks of social evaluation, image damage, or the possibility of being rejected by others; therefore, in stressful situations, they are more likely to choose to avoid or suppress expression [9]. In this study, we focus on a more specific form of disclosure—distress disclosure. Different from the broader concept of self-disclosure, distress disclosure specifically refers to the behavior of individuals actively expressing stress, pain, or negative emotions to others [10]. This type of disclosure not only serves an emotional catharsis function but also promotes psychological recovery through social support and meaning construction. Therefore, understanding the circumstances under which individuals are more willing to engage in distress disclosure holds significant theoretical and practical importance.

In recent years, researchers have begun to focus on the potential positive role of role model information in social comparison. Traditional research has often concentrated on the motivational effects of perfect role models, such as individuals enhancing their self-efficacy and goal pursuit by observing outstanding others [11]. However, overly perfect role models may also create psychological distance and self-threat, leading to feelings of frustration after comparison. In contrast, a new form of role model—imperfect role models—has gradually gained attention. In this study, “imperfect role models” are operationally defined as role model individuals who, while achieving success, also publicly disclose their experiences of failure, setbacks, or struggles. By presenting vulnerability and growth journeys, such role models can enhance observers’ perceived similarity and reduce the threat of social evaluation [12]. When successful individuals display their failure experiences, observers are more likely to generate an assimilation effect, thereby enhancing self-efficacy, reducing defensive reactions, and promoting the willingness to disclose [11].

Nevertheless, individuals’ responses to role model information are not uniform. Some people draw inspiration from it, while others may exhibit defensive reactions, such as denying the value of the role model or belittling their achievements. This variance suggests that personality traits may play a critical moderating role in the process of social comparison. The core personality variable of focus in this study is hostility. In this study, hostility is operationally defined as: an interpersonal cognitive predisposition characterized by suspicion, anger, and defensive interpretation, which makes individuals more likely to interpret others’ behaviors as signals of threat or competition [13]. The reason for selecting hostility as the research variable is that it directly reflects an individual’s defensive tendencies in social interactions. Unlike variables such as growth mindset, which emphasize beliefs about ability, hostility is more closely related to interpersonal defensive responses in social comparison contexts. Existing research indicates that individuals with high hostility are more prone to adopting strategies such as belittling others or denying the value of information in threatening situations to maintain self-consistency [14]. Therefore, when faced with imperfect role models, hostility may influence whether an individual chooses to accept the role model information and engage in self-reflection, or to protect their self-image through defensive denial.

To integrate the aforementioned processes, this study adopts the Meaning Maintenance Model (MMM) as its theoretical framework [15]. MMM posits that humans have a fundamental motivation to maintain consistency within their meaning systems. When individuals experience failure, stress, or threats from social comparison, their existing meaning framework may be disrupted, resulting in psychological discomfort. To restore meaning consistency, individuals initiate a series of meaning repair mechanisms, such as assimilation, accommodation, and affirmation. In the context of social comparison, imperfect role models may help individuals reconstruct their meaning systems by providing a new interpretative framework that allows them to view failure as part of the growth process [16].

Based on this theory, this study proposes that failure-related stress may influence distress disclosure through two distinct pathways. On one hand, when individuals perceive multiple pressures stemming from academic competition, social evaluation, or expectations for future development, their meaning system is threatened, generating psychological tension. Some individuals may protect their meaning through defense mechanisms, such as disparaging the role model or denying their value. On the other hand, when individuals perceive psychological similarity with an imperfect role model, the experience of failure may be reinterpreted as a part of growth, thereby facilitating meaning reconstruction and increasing the willingness to disclose.

To examine this process, this study designed two interconnected sub-studies. Study 1 focuses on the impact of failure stress on distress disclosure and examines the moderating role of hostility in this process, thereby explaining when individuals exhibit defensive reactions when confronted with role model information. Study 2 further explores the mechanism of action of imperfect role models by introducing a scenario involving participation in a “failure experience sharing session” to investigate the psychological processes of individuals when they actively seek out role model information. Specifically, Study 2 will explore the differences in meaning construction among individuals after exposure to various role models, and how this, mediated by their willingness to participate in subsequent sharing sessions, affects distress disclosure. In this context, stress and meaning in life serve as mediators. Here, meaning in life is defined as an individual’s subjective experience of life’s purpose, value, and coherence [17]. Within the MMM framework, meaning in life can be viewed as an important indicator of the restoration of the meaning system.

In summary, this study aims to address three insufficiently answered questions: First, can imperfect role models promote distress disclosure in social comparison contexts, rather than merely triggering self-threat? Second, does an individual’s trait of hostility influence their defensive interpretation of role model information? Third, can the process of meaning reconstruction explain how role model information facilitates individuals’ expression of their failure experiences? By integrating the social comparison perspective with the Meaning Maintenance Model, this study seeks to reveal the psychological mechanism through which individuals transition from “defense” to “meaning reconstruction” under the pressure of failure, and to provide a theoretical basis for promoting authentic self-expression.

## 2. Literature review

### 2.1. Perfect role models vs. imperfect role models

Social role models represent psychological alignment between observers and observed individuals, where people tend to focus on and emulate those who exhibit similar cognitive abilities, behavioral patterns, or social recognition [18]. Models exert both psychological and social influences [19]. Psychologically, they shape self-identity by reflecting discrepancies between individuals’ actual selves and their ideal or “ought” selves. Socially, models disseminate and reinforce norms, encouraging others to modify perceived social standards and imitate their behaviors [20]. For example, peer models can reduce unhealthy behaviors like substance abuse among adolescents [21] or reshape body image expectations [19].

Within social learning frameworks, individuals typically prefer to emulate successful models, which often serve motivational roles in self-improvement [11]. However, contemporary digital spaces are saturated with perfect role models—individuals showcasing curated highlight reels of achievements. These positively biased self-presentations amplify upward social comparisons [22], exacerbating feelings of inadequacy, low self-esteem, anxiety, and depression among observers [23–25], thereby constituting a significant psychological stressor.

Social comparison theory distinguishes between two orientations: (1) traditional social comparisons, which evaluate superiority or inferiority [26], and (2) emotional comparisons, driven by uncertainty about one’s emotional responses [23]. While perfect role models trigger traditional comparisons that foster self-evaluation, self-enhancement, or self-improvement [27,28], emotional comparisons involve horizontal evaluations of emotional states with others in similar situations [23]. The latter reduces uncertainty through shared experiences [29], provides emotional validation [30], and facilitates mutual self-disclosure [31], and serves cognitive clarification and preventive learning functions [23]. Thus, this study argues that imperfect role models—those whose narratives resonate with ordinary struggles—better equip young adults to confront and accept failures [32,33], ultimately encouraging them to share their stories.

### 2.2. The impact of stress on distress disclosure

Stress arises from the interaction between individuals and their environments, representing a subjective response to life challenges after cognitive appraisal. As a major risk factor for mental health, mitigating stress’s adverse effects remains central to contemporary psychological interventions [34]. Intensified competitive pressures (e.g., academic and employment-related “involution”) have heightened stress levels among university students [35,36]. Social comparison theory posits that competitive failures trigger anxiety and stress [23,37–39], reduce self-esteem [40], and provoke defensive withdrawal behaviors [41,42].

Distress disclosure refers to the willingness to express unpleasant emotions to others [43]. It alleviates negative affect by enabling emotional catharsis [44], enhances psychological adaptation and social relationships [45], fulfills belongingness needs [46], strengthens self-awareness, and improves subjective well-being [8]. Disclosure also facilitates social support acquisition [47] and reduces social anxiety [47,48].

However, under stressful life events, individuals often avoid social interactions and suppress negative self-relevant information. According to conservation of resources theory, threats to self-worth in adverse situations diminish perceived social support and deplete psychological resources [49], leading to social avoidance [50]. For instance, students from disadvantaged backgrounds may withdraw socially to avoid potential negative evaluations and protect self-esteem [51]. Suppression theory further indicates that habitual emotional inhibition consumes significant physiological and psychological energy, exacerbating mental health issues [52] like depression [50] and loneliness [53].

In summary, stress inhibits distress disclosure among university students facing failures, thereby worsening mental health outcomes. Since disclosure aids counselors in comprehensively understanding clients’ issues [45], identifying barriers to disclosure and promoting it remain critical research priorities.

**Hypothesis 1:** Stress reduces Distress Disclosure.

### 2.3. The mediating role of self-esteem in the stress-distress disclosure relationship

Self-esteem reflects individuals’ evaluative attitudes toward themselves [54], encompassing affective experiences tied to perceived competence and self-worth [55]. It arises from discrepancies between actual and ideal self-states.

Self-threats—triggered by gaps between real and ideal selves, unfavorable social comparisons, or social exclusion—are ubiquitous [56]. Examples include underperformance at work, upward comparisons with peers’ material success, or workplace ostracism, all of which induce self-threat and psychological distress [57]. The sociometer theory of self-esteem posits that such threats diminish self-esteem [58]. For instance, heightened stress perception among middle school students erodes positive self-assessment foundations, ultimately lowering self-esteem [59].

As a critical psychological resource, self-esteem shapes social experiences and behaviors [60]. Individuals with low self-esteem lack confidence across life domains, hold negative self-views (e.g., regarding social skills, academic ability, appearance), and experience diminished social enjoyment, fostering avoidance, fear, and shame in interpersonal contexts [59]. For example, upward comparisons in social interactions lower self-esteem, triggering low self-esteem, anxiety, and depressive symptoms, which in turn promote social withdrawal to minimize psychological costs [61].

In summary, stress undermines core self-evaluations of competence and worth, reduces self-esteem, and exacerbates negative self-perceptions and social avoidance, ultimately suppressing distress disclosure.

**Hypothesis 2:** Self-esteem mediates the relationship between Stress and Distress Disclosure.

### 2.4. Defense against threat: the moderating role of hostility in the stress-self-esteem relationship

Not all social comparison-induced stress reduces self-esteem. Substantial evidence highlights the protective role of self-affirmation mechanisms: when facing self-threats, individuals may engage in compensatory self-enhancement or affirmations of global self-competence to mitigate negative impacts [61–63].

Maintaining positive self-evaluations and coherent self-concepts constitutes a fundamental human motive, driving threat-resolution behaviors [64]. In order to maintain positive self-evaluations, reduce the perception of pain caused by self-threats, and avoid discrepancies between self-worth and comparison standards, individuals adopt a series of coping behaviors [64], among which hostility represents one such defensive strategy to minimize self-threats and preserve self-worth. However, the defensive mechanism of hostile attribution can lead to many negative consequences, such as restoring damaged self-esteem by belittling others’ worth, which may cause potential role models to be perceived as wasteful or draining [65]. For example, middle school students facing self-esteem threats from stress may develop motivations to protect their self-esteem; these motivations may lead them, out of compensatory psychology, to engage in more destructive aggression to assert their self-worth [59]. Furthermore, self-threats can also heighten interdependence needs, fostering cooperative strategies [66].

Narcissism theory frames hostility as a dynamic self-regulatory process [67]. Because narcissists rely on external validation to maintain self-image, they disproportionately attribute hostility to threat sources (e.g., devaluing critical evaluators) to neutralize self-threats [68]. Workplace studies corroborate this: employees making upward comparisons often derogate targets (e.g., criticizing or belittling peers) to alleviate self-threats [69].

In summary, narcissistic individuals leverage hostility to buffer self-esteem against stress-induced threats, thereby attenuating the detrimental effects of social comparisons [61].

**Hypothesis 3:** High-hostility weakens the negative impact of Stress on Self-esteem, thereby reducing the mediating effect of Self-esteem on Distress Disclosure. Conversely, Low-hostility strengthens this mediation.

### 2.5. Threat reconstruction: The mediating role of meaning in life

When direct resolution of self-threats is unfeasible, cognitive reconstruction offers an alternative pathway. For instance, Wan et al. found that individuals facing prolonged social exclusion may reinterpret rejection as evidence of their uniqueness, thereby preserving self-integrity [70].

Exposure to imperfect role models necessitates confronting personal failures and their parallels with others’ experiences, which activates stress responses. Stress further erodes meaning in life by undermining basic psychological needs (autonomy, competence, relatedness) [17].

Meaning in life denotes the extent to which individuals comprehend or perceive purpose, missions, and overarching goals in their existence [17]. The meaning maintenance model (MMM) posits that events violating preexisting beliefs (e.g., failures, contradictory information) trigger meaning violations, eliciting stress responses (e.g., anxiety, cognitive dissonance). To alleviate this distress, individuals engage in meaning-making efforts to restore coherence—for example, downplaying failure significance or reorienting toward familial goals [71].

Meaning in life reframes painful experiences into “coherent growth narratives.” Individuals with high meaning in life view disclosure as a therapeutic and relational act rather than a risk. By reconstructing failure (e.g., “setbacks build resilience”), they legitimize emotional expression, overcome shame, and initiate disclosure. Enhanced meaning in life promotes distress disclosure by providing psychological resources and interpretive frameworks that encourage confronting rather than avoiding adversity [71].

**Hypothesis 4:** Stress and Meaning in Life sequentially mediate the relationship between Participation Willingness and Distress Disclosure.

This mediation is significant only in the adversity perception group, indicating that stress-driven meaning reconstruction dominates when individuals perceive others’ experiences as adversities. In contrast, stress merely reflects pure threat in prosperity perception contexts, suppressing meaning and disclosure.

**Hypothesis 5:** An individual’s situational perception of whether another person’s experience is perceived as an advantage (prosperity) or a disadvantage (adversity) moderates the relationship between participation willingness and distress disclosure. Specifically, in the perceived prosperity group, participation willingness inhibits distress disclosure, whereas in the perceived adversity group, participation willingness promotes distress disclosure.

## 3. Methodology

### 3.1. Study1

#### 3.1.1. Participants and procedure.

This study was approved by the Ethics Committee of Changjiang College. Informed consent was obtained from the respondents before they filled out the questionnaire. A failure-sharing session was convened in which a pre-arranged student shared their own failure experiences, describing how they overcame various failures and setbacks and eventually achieved success. Participants were invited to attend the session as listeners. Data were collected through an online questionnaire distributed at the session.

A total of 218 undergraduate students who viewed the role models as Imperfect Models were recruited, including 51 (23.4%) males and 167 (76.6%) females (Mean age = 19.59 years, SD = 1.16 years).

In this failure-sharing session, the researchers presented the students with a story like this: “Student A had outstanding academic performance at university. Although they failed in the first National College Student Mathematical Modeling Competition, they summarized their failures and won first prize in the second competition. As Vice President of the Student Union, Student A organized multiple campus cultural activities, enduring many hardships in the process. Student A also participated in research projects under a supervisor, and after multiple attempts and failures, finally published an academic paper. Near graduation, Student A sought both postgraduate studies and employment but unfortunately failed in both. Student A will share the pitfalls and failure experiences of college life at a graduate experience-sharing session.”

Given the failure scenario induction (e.g., negative experiences of imperfect role models) in this study, a standardized debriefing was immediately conducted post-experiment: participants were explicitly informed that “Student A’s story” was fictionally constructed with experimental manipulations, not reflecting universal outcomes, to alleviate situational anxiety about academics or employment. Multiple mental health support measures were implemented, including remuneration for participation, on-site emotional support from psychology-trained research assistants, and access to free counseling (contact provided in the questionnaire). Observation records confirmed all participants departed with stable emotions and no significant psychological distress.

#### 3.1.2. Research tools.

When selecting scales, this study prioritized those published in high-level domestic and international journals with good reliability and validity. Foreign language scales were translated through a strict bidirectional translation process, participants gave written informed consent.

**Distress Disclosure Index (DDI)**: Distress Disclosure was measured using the Distress Disclosure Index (DDI). The Distress Disclosure Scale developed by Kahn and Hessling was used to measure the degree to which individuals disclose their pain, worries, and other private matters to others [10]. It consists of 12 items using a 5-point Likert scale from “completely inconsistent” (1) to “completely consistent” (5). After reverse scoring the negatively worded items, the average score was calculated by summing the item scores and then dividing by the number of items. Higher scores indicate a stronger tendency toward self-disclosure and a greater inclination to share negative personal information with others, while lower scores indicate a greater tendency to conceal negative personal information from others. Sample items include: “I prefer not to talk about my problems” and “When I am in a bad mood, I talk about it with my friends.” The Cronbach’s α coefficient for the original English version was 0.94. The scale was translated into a Chinese version for this study using multiple rounds of back-translation. The final Chinese version was pretested on a sample of 40 participants to examine item clarity and comprehensibility. Based on feedback, all items were clear and understandable, and no further revisions were needed. In this study, the Cronbach’s α coefficient for the translated scale was 0.91.

**Narcissistic Admiration and Rivalry Questionnaire (NARQ)**: The measurement scale for the hostility variable was derived from the hostility subscale of the Narcissistic Admiration and Rivalry Questionnaire (NARQ) developed by Back et al. [14]. This study used the Chinese version translated by Deng et al. [72]. This questionnaire consists of 18 items. Items 1–9 measure admiration (e.g., “I deserve to be worshipped as an idol by others” or “I revel in the achievements I have attained.”), while items 10–18 measure rivalry (e.g., “When criticized by others, I get very annoyed.” or “Except for me, other people have no real value.”). In the original study by Back (2013), the Cronbach’s α coefficient for the overall scale was 0.80, with 0.84 for the admiration dimension and 0.80 for the rivalry dimension; the Cronbach’s α coefficient for the hostility subscale was 0.66 [14]. The Chinese version of the scale used in this study had a Cronbach’s α coefficient of 0.86 for the overall scale, 0.84 for the admiration dimension, and 0.80 for the rivalry dimension, though reliability coefficients for the sub-dimensions were not reported. The scale uses a 6-point Likert scale (1 = “completely inconsistent” to 6 = “completely consistent”), and the total score is the sum of the scores for each item. Higher scores indicate higher levels of admiration and rivalry. Only the hostility subscale was selected in this study. The Cronbach’s α coefficient for the overall scale in this study was 0.83, with 0.82 for the admiration dimension and 0.85 for the rivalry dimension; the Cronbach’s α coefficient for the hostility subscale used in this study was 0.69.

**Depression Anxiety and Stress Scale-21**: The measurement of the stress variable was selected from the abbreviated version of the Depression Anxiety and Stress Scale-21 (DASS-21), which was adapted from the 42-item original version developed by Lovibond et al [73]. The simplified Chinese version of the DASS-21 revised by Gong Xu et al. [74] was used in this study. In the original 42-item version, the Cronbach’s α coefficients were 0.91 for the Depression subscale, 0.84 for the Anxiety subscale, and 0.90 for the Stress subscale, with no reliability coefficient reported for the total scale. The Chinese version by Gong et al. has demonstrated good reliability, with a Cronbach’s α of 0.89 for the total scale, and 0.77, 0.79, and 0.76 for the Depression, Anxiety, and Stress subscales, respectively. The scale consists of 21 items, with each of the three subscales—Depression (DASS-D), Anxiety (DASS-A), and Stress (DASS-S)—containing 7 items. Example items include “It seems that I no longer have any pleasant or comfortable feelings” for the Depression subscale, “I worry about situations that might make me panic or embarrass myself” for the Anxiety subscale, and “I cannot tolerate anything that prevents me from continuing my work” for the Stress subscale. A 4-point Likert scale is employed (ranging from 1 = “completely inconsistent” to 4 = “completely consistent”), where higher scores indicate stronger levels of the corresponding emotion. Only the stress subscale was used in this study. The Cronbach’s α coefficient for the overall scale in this study was 0.93, with coefficients of 0.82 for the Stress subscale, 0.82 for the Anxiety subscale, and 0.83 for the Depression subscale.

**Rosenberg’s Self-Esteem Scale**: The measurement of state self-esteem adopted the Chinese version of the Rosenberg Self-Esteem Scale [75], originally developed by Rosenberg et al [76]. The Cronbach’s α coefficient of the original scale was 0.88, and that of the Chinese version was 0.87. It consists of 10 items(e.g., “At this moment, I hold a positive attitude toward myself” and “At this moment, I can do things as well as most people”)rated on a 4-point Likert scale (1 = “completely inconsistent” to 4 = “completely consistent”). After reverse-scoring the negatively worded items, a higher total score indicates a higher level of self-esteem among participants. The Cronbach’s α coefficient of this scale was 0.85.

#### 3.1.3. Statistical analysis.

Data analysis was conducted using SPSS 21.0. Data are presented as mean and standard deviation (Mean ± SD). Pearson’s correlation analysis was used to explore correlations between variables at the 0.05 significance level. Harman’s single-factor test was employed to determine whether common method bias existed in the study. Hypothesis testing was conducted using Hayes’ PROCESS macro, with Model 4 for mediation analysis and Model 7 for moderated mediation analysis.

### 3.2. Study 2

#### 3.2.1. Participants and procedure.

Study 2 was a situational experiment involving undergraduate students. The experiment was approved by the Ethics Committee of Yangtze College. Informed consent was obtained from the respondents before they filled out the questionnaire. Based on the effect size (Cohen’s d = 0.4) and expected power (Power = 0.8), G*Power 3.1 was used to calculate the planned sample size of 200. After screening, 536 valid responses were obtained in Study 2, including 119 males (22.2%) and 417 females (77.8%), with an average age of 19.60 years (SD = 1.27).

#### 3.2.2. Experimental design and procedure.

Study 2 employed a single-factor between-subjects experimental design. Participants were randomly assigned to either the “success” condition (N = 268, 50%) or the “failure” condition (N = 268, 50%) using the “random scenario” function of Wenjuanxing to activate subsequent psychological responses. Among them, 303 participants (56.5%) perceived the scenario as a prosperity, 233 (43.5%) regarded the story in the sharing session as adversity and regarded it as an imperfect role model, and 459 (85.6%) expressed willingness to participate in the success/failure experience sharing session (0 = willing to participate), while 77 (14.4%) were unwilling (1 = unwilling to participate). There were no significant demographic differences between the two groups (p > 0.05).

After the experiment began, participants first completed demographic information. They then read a passage about a student’s successes and struggles in school (see below) and were instructed to imagine the experience as real. After reading, participants recalled and described the content, then sequentially answered manipulation check items, the Participation Willingness, the abbreviated Depression Anxiety and Stress Scale-21 (DASS-21), Self-Esteem Scale, Distress Disclosure Index, Narcissistic Admiration and Rivalry Questionnaire, and Meaning in Life Scale.

Given the failure scenario induction (e.g., negative experiences of imperfect role models) in this study, a standardized debriefing was immediately conducted post-experiment: participants were explicitly informed that “Student A’s story” was fictionally constructed with experimental manipulations, not reflecting universal outcomes, to alleviate situational anxiety about academics or employment. Multiple mental health support measures were implemented, including remuneration for participation, on-site emotional support from psychology-trained research assistants, and access to free counseling (contact provided in the questionnaire). Observation records confirmed all participants departed with stable emotions and no significant psychological distress.

**Scenario Materials**: The experimental materials were developed by the researchers based on personal experiences and real-world conditions.

**Success Scenario Group**: Participants read the following: “Student A achieved excellent academic performance at university, winning the first prize in the National College Student Mathematical Modeling Competition. Student A actively engaged in student leadership roles, serving as Vice President of the Student Union, organizing and planning multiple campus cultural activities, and was highly praised by teachers and students. Additionally, Student A participated in research projects under a supervisor, gained extensive research experience, and published an academic paper. Near graduation, Student A pursued both postgraduate entrance exams and job applications simultaneously and succeeded in both. Student A will share successful college life experiences at a graduate experience-sharing session. "

**Failure Scenario Group**: Participants read the following: “Student A had outstanding academic performance at university. Although they failed in the first National College Student Mathematical Modeling Competition, they summarized their failures and won first prize in the second competition. As Vice President of the Student Union, Student A organized multiple campus cultural activities, enduring many hardships in the process. Student A also participated in research projects under a supervisor, and after multiple attempts and failures, finally published an academic paper. Near graduation, Student A sought both postgraduate studies and employment but unfortunately failed in both. Student A will share the pitfalls and failure experiences of college life at a graduate experience-sharing session.”

#### 3.2.3. Measurement tools.

Data analysis was performed using SPSS 21.0 and AMOS 24.0. When selecting scales, this study prioritized instruments published in high-quality domestic and international journals with established reliability and validity. Foreign language scales were translated through a rigorous bidirectional translation process.

**Participation Willingness**: Participation Willingness was measured using a two-point scale item: “Based on your personal feelings, if time permits, would you be willing to participate in Student A’s experience-sharing session?” (0 = willing to participate, 1 = unwilling to participate).

**Distress Disclosure Index (DDI)**: Distress Disclosure was measured using the Distress Disclosure Index (DDI). The Distress Disclosure Scale developed by Kahn and Hessling was used to measure the degree to which individuals disclose their pain, worries, and other private matters to others [10]. It consists of 12 items using a 5-point Likert scale (from 1 = “completely inconsistent” to 5 = “completely consistent”). After reverse scoring the negatively worded items, the average score was calculated by summing the item scores and then dividing by the number of items. Higher scores indicate a stronger tendency toward self-disclosure and a greater inclination to share negative personal information with others, while lower scores indicate a greater tendency to conceal negative personal information from others. Sample items include: “I prefer not to talk about my problems” and “When I am in a bad mood, I talk about it with my friends.” The Cronbach’s α coefficient for the original English version was 0.94. The scale was translated into a Chinese version for this study using multiple rounds of back-translation. The final Chinese version was pretested on a sample of 40 participants to examine item clarity and comprehensibility. Based on feedback, all items were clear and understandable, and no further revisions were needed. In this study, the Cronbach’s α coefficient for the translated scale was 0.91.

**Depression Anxiety and Stress Scale-21**: The measurement of the stress variable was selected from the abbreviated version of the Depression Anxiety and Stress Scale-21 (DASS-21), which was adapted from the 42-item original version developed by Lovibond et al. [73]. The simplified Chinese version of the DASS-21 revised by Gong Xu et al. [74] was used in this study. In the original 42-item version, the Cronbach’s α coefficients were 0.91 for the Depression subscale, 0.84 for the Anxiety subscale, and 0.90 for the Stress subscale, with no reliability coefficient reported for the total scale. The Chinese version by Gong et al. has demonstrated good reliability, with a Cronbach’s α of 0.89 for the total scale, and 0.77, 0.79, and 0.76 for the Depression, Anxiety, and Stress subscales, respectively. The scale consists of 21 items, with each of the three subscales—Depression (DASS-D), Anxiety (DASS-A), and Stress (DASS-S)—containing 7 items. Example items include “It seems that I no longer have any pleasant or comfortable feelings” for the Depression subscale, “I worry about situations that might make me panic or embarrass myself” for the Anxiety subscale, and “I cannot tolerate anything that prevents me from continuing my work” for the Stress subscale. A 4-point Likert scale is employed (ranging from 1 = “completely inconsistent” to 4 = “completely consistent”), where higher scores indicate stronger levels of the corresponding emotion. Only the stress subscale was used in this study. The Cronbach’s α coefficient for the overall scale in this study was 0.93, with coefficients of 0.80for the Stress subscale, 0.83for the Anxiety subscale, and 0.83 for the Depression subscale.

**Meaning in Life Questionnaire (MLQ)**: Meaning in Life was assessed using the Meaning in Life Questionnaire (MLQ) developed by Steger et al. [17]and revised by Wang [77]. The scale includes 10 items across two subscales: Presence of Meaning and Search for Meaning. Sample items include “My life has no clear purpose,” “I understand the meaning of my life” (Presence), and “I am seeking a purpose or mission in my life,” “I am searching for the meaning of my own life” (Search). The original scale by Steger et al. (2006) reported Cronbach’s α coefficients of 0.86 for Presence and 0.87 for Search, but did not report overall reliability. The Chinese version had Cronbach’s α coefficients of 0.84 for the total scale, 0.79 for Presence, and 0.78 for Search. Using a 7-point Likert scale (1 = “completely inconsistent” to 7 = “completely consistent”), higher scores indicate stronger meaning in life. In the present study, the overall scale had a Cronbach’s α coefficient of 0.84, with the Presence subscale at 0.84 and the Search subscale at 0.86.

## 4. Results

### 4.1. Study 1

#### 4.1.1. Common method bias test.

Harman’s single-factor test was used to examine common method bias. Results showed 7 factors with eigenvalues >1, with the first factor explaining 26.69% of variance (below the 40% threshold), indicating no severe common method bias.

#### 4.1.2. Correlation analysis.

Descriptive statistical analyses of the means, standard deviations, and correlations among the main variables in this study were conducted using SPSS 21.0 software. The average scores and correlation results for each variable are presented in Table 1. The mean scores for Stress (M = 0.04, SD = 0.83), State Self-Esteem (M = 0.15, SD = 0.93), and Distress Disclosure (M = 0.11, SD = 1.10) were all above the theoretical median of 0, indicating that the participant group exhibited higher levels of stress, self-esteem, and distress disclosure. In contrast, the mean score for Hostility (M = −0.13, SD = 0.91) was below the theoretical median of 0, suggesting lower hostility levels among participants. Additionally, Stress was negatively correlated with State Self-Esteem and Distress Disclosure; Hostility was negatively correlated with both State Self-Esteem and Distress Disclosure; and Hostility was positively correlated with Stress, while State Self-Esteem was positively correlated with Distress Disclosure.

**Table 1:** Descriptive statistics of the latent constructs.

	M ± SD	1	2	3	4
**1 Stress**	0.04 ± 0.83	–			
**2 State Self-Esteem**	0.15 ± 0.93	−0.56^**^	–		
**3 Distress Disclosure**	0.11 ± 1.10	−0.28^**^	0.32^**^	–	
**4 Hostility**	−0.13 ± 0.91	0.37^**^	−0.34^**^	−0.15^**^	–

Note: n = 218; values in the table are calculated as Z-scores, ^**^: p < 0.01.

#### 4.1.3. Moderated mediation analysis.

First, the mechanism of how Stress affects Distress Disclosure was tested with State Self-Esteem as the mediator variable, results in Table 2. Mediation effect analysis was conducted using Model 4 in the PROCESS program of SPSS 21.0. The bias-corrected percentile Bootstrap method was used to test the confidence interval (CI) estimation, with 5,000 resampling iterations to calculate the 95% CI. The results showed that Stress had a direct predictive effect on Distress Disclosure (β = −0.37, t = −4.31, p < 0.001), supporting H1. Additionally, Bootstrap test results indicated that the 95% CIs of both the direct and indirect effects of State Self-Esteem did not include 0 (Table 3), suggesting that State Self-Esteem played a partial mediating role in the relationship between Stress and Distress Disclosure. The partial mediation effect was −0.17, accounting for 45.95% of the total effect, supporting H2.

**Table 2:** Mediation Model Test of Self-Esteem.

Regression Equation		Fitting indices		Coefficient significance		
outcome	Predictor	R	R2	F	β	t
Distress Disclosure	Stress	0.28	0.08	18.59***	−0.37	−4.31
State Self-Esteem	Stress	0.55	0.31	96.09***	−0.62	−9.80
Distress Disclosure	Stress	0.34	0.12	14.15***	−0.20	−1.99
	State Self-Esteem				0.27	3.00

Note: n = 218; all variables in the model were standardized; ***: p < 0.001.

**Table 3:** Total, Direct, and Indirect Effects of Self-Esteem Between Stress and Distress Disclosure.

Effect Type	Effect Value	Boot SE	95%CI
Total Effect	−0.37	0.09	[-0.54,-0.20]
Direct Effect	−0.20	0.10	[-0.40,-0.00]
Indirect Effect	−0.17	0.07	[-0.31,-0.04]

**Fig 1:**
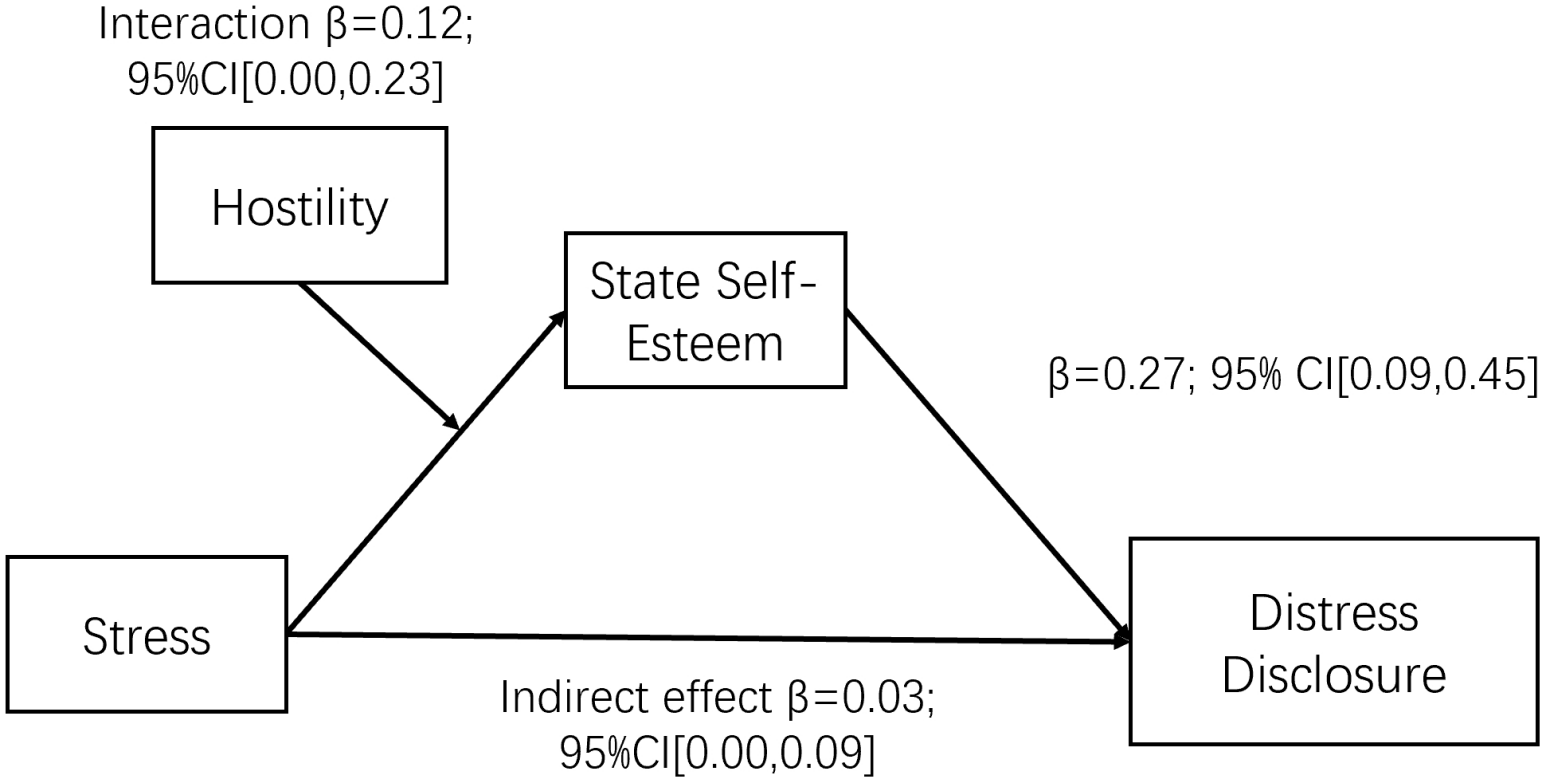
Moderated Mediation Effect of Self-Esteem and Hostility in the Influence of Stress on Distress Disclosure.

Moderated mediation analysis was conducted using Model 7 in the PROCESS program of SPSS 21.0, with standardized path coefficients shown in Fig 1. This model assumes that the moderator variable can moderate the front pathway of the mediation model, consistent with the study’s hypothesis. Results (Tables 4 and 5) showed that the interaction term of Stress and Hostility had a statistically significant effect on State Self-Esteem (β = 0.12, p < 0.05).Moreover, the moderating effect of Hostility on the mediating pathway through self-esteem also reached statistical significance (Index = 0.03, SE = 0.02, CI=[0.00, 0.09]), supporting Hypothesis 3.

**Table 4:** Moderated Mediation Model Test.

Predictors	Model1(SE)			Model2(DD)		
	β	t	95%CI	β	t	95%CI
Stress	−0.54	−8.13^***^	[-0.68,-0.41]	−0.20	−1.99^*^	[-0.40,-0.00]
State Self-Esteem				0.27	3.00^***^	[0.09,0.45]
Stress × Hostility	0.12	2.01^*^	[0.00,0.23]			
Hostility	−0.16	−2.64^**^	[-0.28,-0.04]			
R2	0.34			0.12		
F	37.05^***^			14.15^***^		

Note: n = 218; all variables in the model were standardized; ^*^: p < 0.05; ^**^: p < 0.01; ^**^: p < 0.001; SE(self-esteem), DD(distress disclosure).

**Table 5:** Mediation Effects of Self-Esteem at Different Levels of Hostility.

Moderator	Standardized Mediation Effect	SE	95%CI
Low (M-1SD)	−0.18	0.08	[-0.345,-0.04]
Medium (M)	−0.15	0.06	[-0.28,-0.04]
High (M + 1SD)	−0.12	0.05	[-0.24,-0.03]
Moderated Mediation Effect	0.03	0.02	[0.00,0.09]

**Fig 2:**
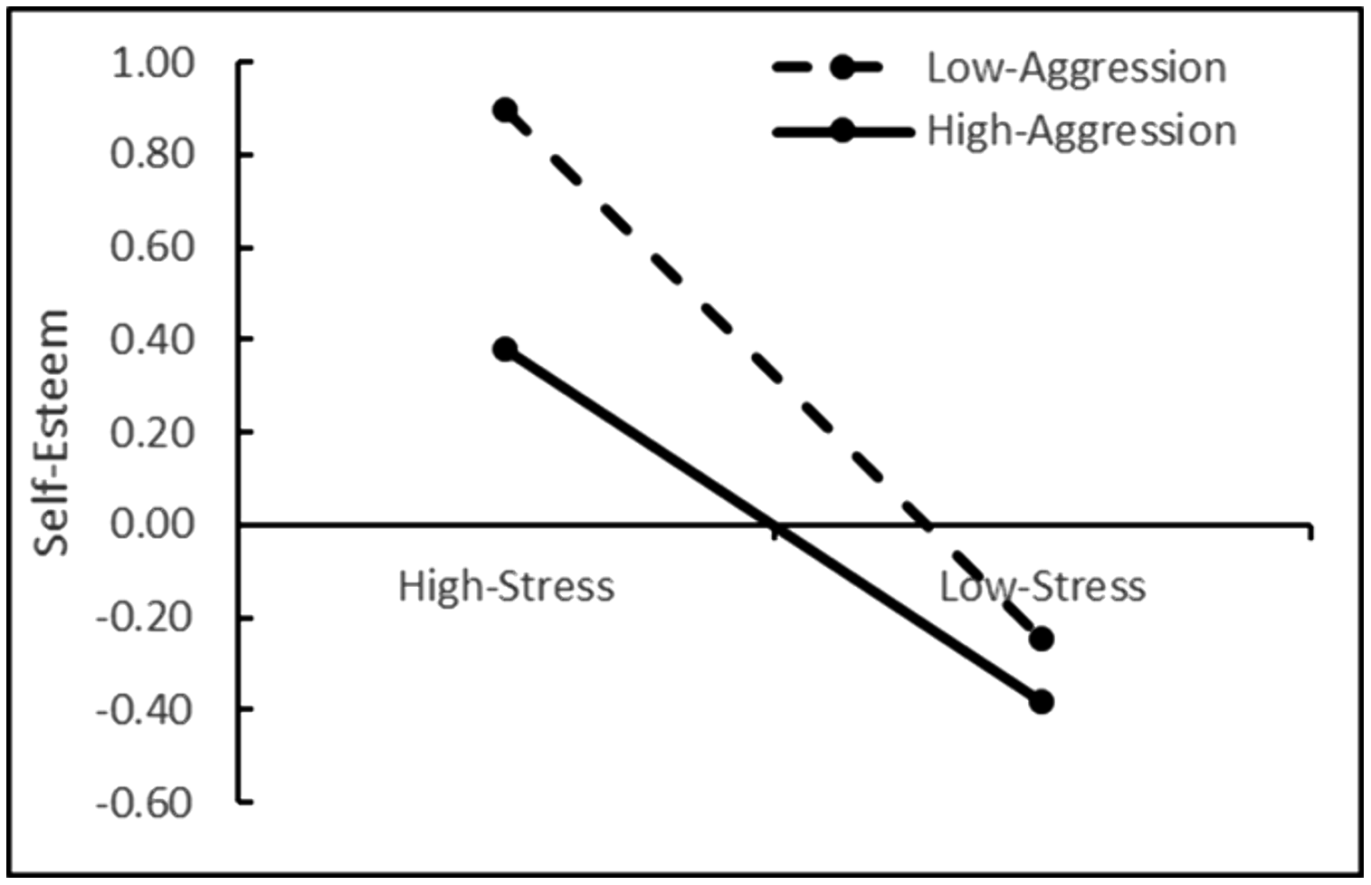
Moderating Effect of Hostility on the Relationship Between Stress and State Self-Esteem.

To further reveal the nature of the interaction effect between Stress and Hostility, a simple effects plot was used to analyze the moderating role of Hostility. Hostility was divided into high (M + 1SD) and low (M-1SD) groups for simple slope analysis. The results showed that in both high Hostility (β = −0.45, t = −5.43, p < 0.001) and low Hostility (β = −0.67, t = −7.53, p < 0.001) conditions, State Self-Esteem showed a significant downward trend as Stress increased (Fig 2), and this trend was mitigated (with a smaller slope) at higher Hostility levels, supporting H3.

### 4.2. Study 2

#### 4.2.1. Manipulation check.

In the set of manipulation check questions, participants were asked whether the material involved academic performance, student council, and research activities. All participants reported that the material covered these topics, indicating that they had carefully read and understood the story presented in the material.

To examine the association between experimental manipulation (failure vs. success condition) and perceived grouping (adversity perception vs. prosperity perception), thereby verifying the effectiveness of the experimental manipulation, a 2 × 2 contingency table chi-square test was conducted on the data. Results showed a highly significant association between experimental manipulation and perceived grouping, Pearson χ²(1, N = 536) = 312.87, p < 0.001, with an effect size of φ = 0.76 (strong effect). Assumption checks indicated that all expected cell frequencies were≥5 (minimum expected count = 116.50), satisfying the assumptions for Pearson’s chi-square test and confirming the reliability of the results. Further analysis of within-group proportions revealed that in the failure condition, 89.2% of participants perceived the situation as adversity, while only 10.8% perceived it as prosperity. In contrast, in the success condition, 87.5% of participants perceived the situation as prosperity, while only 12.5% perceived it as adversity. These findings indicate that the experimental manipulation successfully induced the intended differences in participants’ situational perceptions, providing a reliable foundation for subsequent hypothesis testing.

To test whether the experimental manipulation had a confounding effect on participation willingness, a 2 × 2 chi-square test was conducted to analyze the association between random assignment (A’s success scenario vs. A’s failure scenario) and participants’ willingness to participate (willing vs. unwilling). Results showed no significant association between the two variables, χ²(1, N = 536) = 0.14, p = 0.712, φ = −0.016. Assumption checks confirmed that all expected cell frequencies were≥5 (minimum expected count = 38.50), satisfying the assumptions for Pearson’s chi-square test and ensuring the reliability of the results. Specifically, in the success scenario group, 85.1% of participants expressed willingness to attend the experience-sharing session, while 14.9% were unwilling. In the failure scenario group, 86.2% of participants expressed willingness, while 13.8% were unwilling. The proportions of participation willingness were highly comparable between the two groups. This result suggests that the experimental manipulation did not significantly affect participants’ willingness to participate, ruling out participation willingness as a confounding variable and providing a reliable basis for the subsequent mediation analysis.

#### 4.2.2. Common method bias test.

To assess common method bias, a Harman single-factor test was conducted. The results revealed that there were 5 factors with eigenvalues greater than 1, and the first factor accounted for 25.43% of the variance, which is below the critical threshold of 40%. Therefore, no severe common method bias was detected in this study.

#### 4.2.3. Descriptive statistics.

The mean scores of participants on each variable in this study and the results of correlation analysis are shown in Table 6. The mean scores of Stress (M = 0.11, SD = 0.81), Sense of Life Meaning (M = 0.00, SD = 1.00), and Distress Disclosure (M = 0.11, SD = 1.07) were all higher than the theoretical median of 0, indicating that the participant group had higher levels of stress, sense of life meaning, and distress disclosure. In addition, stress was negatively correlated with both sense of life meaning and distress disclosure, while sense of life meaning was positively correlated with distress disclosure.

**Table 6:** Standardized Descriptive Statistics and Pearson Correlation Analysis Results.

	M ± SD	1	2	3
1 Stress	0.11 ± 0.81	–		
2 Meaning in Life	0.00 ± 1.00	−0.27^***^	–	
3 Distress Disclosure	0.11 ± 1.07	−0.25^***^	0.28^***^	–

Note: n = 536; ^*^: p < 0.05; ^**^: p < 0.01; ^***^: p < 0.001.

It should be noted that the categorical variable “Participation Willingness” is used only for subsequent group comparisons and path analysis; means and standard deviations were not calculated. Its distribution is detailed in the aforementioned Participants section (or via frequency summary).

#### 4.2.4. Chain mediation model test.

First, to examine the mechanism of how stress influences distress disclosure, self-esteem was tested as a mediator in Table 7. Chain mediation analysis was conducted using Model 6 in the PROCESS program. The bias-corrected percentile Bootstrap method (5,000 resampling iterations) was used to estimate 95% confidence intervals (CIs). The results (Table 7) showed that participation willingness did not directly predict distress disclosure (β = −0.17, t = −1.33, p > 0.05). However, participation willingness positively predicted stress (β = 0.23, t = 2.33, p < 0.05) and negatively predicted meaning in life (β = −0.52, t = −4.40, p < 0.001). Stress negatively predicted meaning in life (β = −0.31, t = −6.07, p < 0.001) and distress disclosure (β = −0.25, t = −4.40, p < 0.001), while meaning in life positively predicted distress disclosure (β = 0.24, t = 5.10, p < 0.001). Bootstrap results showed that the 95% CIs of the indirect effects (via stress and meaning in life) and the total indirect effect did not include 0 (Table 8), indicating that stress and meaning in life fully mediated the relationship between participation willingness and distress disclosure. The total mediation effect was −0.20, accounting for 54.05% of the total effect, supporting H4.

**Table 7:** Chain Mediation Model Test.

Regression Equation		Fitting indices			Coefficient significance	
Outcome	Predictor	R	R2	F	β	t
S	PW	0.10	0.01	5.42	0.23	2.33^*^
MIL	PW	0.32	0.10	31.07	−0.52	−4.40^***^
	S				−0.31	−6.07^***^
DD	PW	0.34	0.12	23.10	−0.17	−1.33
	S				−0.25	−4.40^***^
	MIL				0.24	5.10^***^

Note: n = 536; all variables were standardized; ^*^:p < 0.05; ^**^:p < 0.01; ^***^:p < 0.001; PW(Participation Willingness), S(Stress), MIL(Meaning in Life), DD(Distress Disclosure).

**Table 8:** Total, Direct, and Mediation Effects of Chain Mediation.

Effect Type	Trials	Effect Size	Boot SE	95%CI
ind1	PW → S → DD	−0.06	0.03	[-0.12,-0.01]
ind2	PW → MIL → DD	−0.12	0.04	[-0.21,-0.05]
ind3	PW → S → MIL → DD	−0.02	0.01	[-0.04,-0.00]
totind	ind1 + ind2 + ind3	−0.20	0.06	[-0.32,-0.09]
c	PW → DD	−0.17	0.13	[-0.42,0.08]
total	totind+c	−0.36	0.13	[-0.62,-0.11]

Note: PW(Participation Willingness), S(Stress), MIL(Meaning in Life), DD(Distress Disclosure).

#### 4.2.5. Cross-group comparison of moderated mediation effects.

To test whether the chain mediation model differed between the two experimental contexts, this study conducted multi-group path analysis to examine whether participants’ perception of others’ experiences as prosperous or adverse circumstances would affect their subsequent distress disclosure, and whether the mediating roles of self-esteem and meaning in life remained valid in this process. Since this model was a saturated model—i.e., all parameters to be estimated exactly equaled the elements in the covariance matrix, with degrees of freedom df = 0—fit indices were not estimated, and only path coefficients were focused on [67].

**Fig 3:**
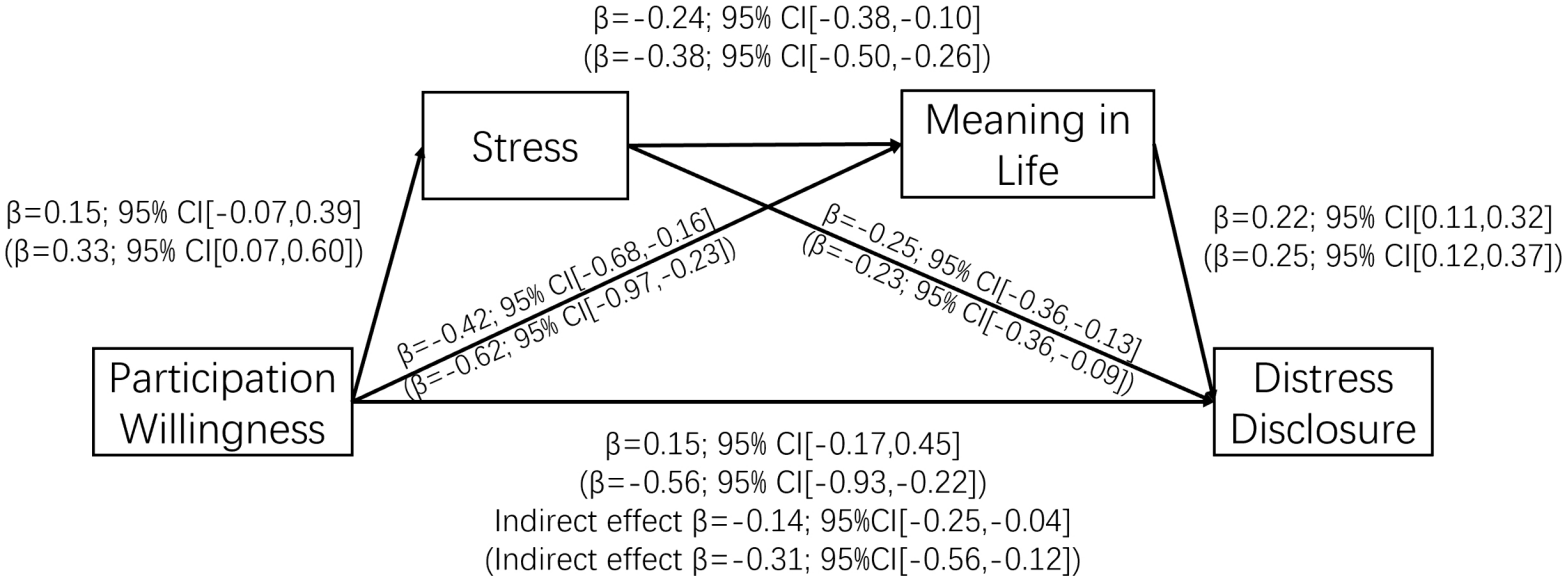
Chain Mediating Effects of Stress and Meaning in Life Between Participation Willingness and Distress Disclosure. Note: Path coefficients outside parentheses are for the prosperous circumstance perception group, and those inside are for the adverse circumstance perception group; *: p < 0.05; **: p < 0.01; **: p < 0.001; PW(Participation Willingness), S(Stress), MIL(Meaning in Life), DD(Distress Disclosure).

Further comparisons were made to determine whether differences in structural equation model path coefficients between the two groups were statistically significant. Standardized path coefficients of the mediation models in both groups are shown in Fig 3. The critical ratio (CR) of parameter differences was used to compare regression coefficients of the structural model paths across groups. Results showed that in the prosperous-circumstance perception group, the 95% confidence intervals (CIs) of the mediation effects of stress and meaning in life, as well as the chain mediation effect, all included 0, indicating nonsignificant mediation effects. In the adverse-circumstance perception group, the 95% CIs of these mediation effects did not include 0, and the main effect of participation willingness on distress disclosure was significant, with all partial mediation effects being significant.

Given that the hypothesized chain mediation model is saturated, the unconstrained model yielded df = 0 and CMIN = 0, shifting the focus of model comparison from global fit indices to specific parameter differences. Subsequent restricted models exhibited excellent fit to the data, providing a rigorous statistical basis for comparing path coefficients via critical ratio (CR) tests.

Through CR tests (where an absolute value >1.96 indicates a significant parameter difference) [78], it was found that except for the path coefficient of participation willingness on distress disclosure (β = 0.15 in the prosperous group vs.β = −0.56 in the adverse group, CR = −2.79), no significant differences existed in other path coefficients between the two groups (Table 9). This suggests that when individuals perceive others’ shared experiences as adverse circumstances, the process of increased stress followed by decreased meaning in life is more likely to trigger willingness to disclose distress.

**Table 9:** Comparison of CI Values for Each Path.

Independent Variable	Dependent Variable	CI
Participation Willingness	Stress	0.88
Stress	Meaning in Life	−1.36
Meaning in Life	Distress Disclosure	0.30
Participation Willingness	Meaning in Life	0.14
Stress	Distress Disclosure	−0.82
Participation Willingness	Distress Disclosure	−2.79

The multi-group path analysis revealed that the chain mediation model exhibited different patterns of effects under different situational perceptions. Under the condition where others’ experiences were perceived as prosperous, the effect of participation willingness on distress disclosure operated primarily through the indirect paths of stress and meaning in life, with no significant direct effect, manifesting as a full mediation model. In contrast, under the condition where others’ experiences were perceived as adverse, participation willingness not only influenced distress disclosure through the chain mediation of stress and meaning in life but also had a significant direct effect, presenting as a partial mediation model. Furthermore, the multi-group comparison results showed a significant difference between the two groups in the direct path from participation willingness to distress disclosure, indicating that in the adversity perception context, participation willingness has a more direct and significant facilitating effect on individuals’ distress disclosure. In other words, compared to those unwilling to participate in the imperfect role model experience sharing session, or those facing the perfect role model sharing session, individuals more willing to participate in the imperfect role model session disclosed more self-related negative information, supporting H5.

## 5. Discussion

### 5.1. Study 1: The inhibitory effect of stress on self-disclosure and the moderating role of hostility

Data from Study 1 indicated that stress had a significant inhibitory effect on distress disclosure. In the direct path, stress significantly suppressed distress disclosure (β = −0.37, p < 0.001). Meanwhile, indirect path analysis revealed that stress further inhibited distress disclosure by lowering self-esteem (β_indirect = −0.17, 95% CI=[−0.31, −0.04]). Moderated mediation analysis found a significant moderating effect of the hostility on the relationship between stress and self-esteem (β_index = 0.03, 95% CI=[0.0004, 0.0929]).

The significance of the direct path indicates that when individuals face stress, their self-defense mechanisms may be activated, thereby reducing the tendency to actively express inner pain. The significance of the mediation effect suggests that stress is not merely an external event but also a process of self-meaning construction. This mechanism complements the explanatory framework of social comparison theory from the perspective of self-system impairment, suggesting that an individual’s distress disclosure behavior under stress is influenced both by the assessment of external threats and constrained by the level of self-esteem. The significance of the mediated moderation effect indicates that individuals with different personality traits may employ distinct psychological defense pathways when facing stress. Specifically, among individuals with high hostility, the negative impact of stress on self-esteem was significantly attenuated, suggesting that they may possess certain self-protective mechanisms when confronting threats, thereby maintaining a relatively stable level of self-evaluation. In contrast, individuals with low hostility were more susceptible to the impact of stress, experiencing a greater decrease in self-esteem.

In summary, the results of Study 1 indicate that when individuals are confronted with an imperfect model, stress exerts a dual inhibitory effect on distress disclosure: it both directly inhibits disclosure through threat and indirectly inhibits it by lowering self-esteem. This provides empirical evidence for understanding individuals’ disclosure decisions in social contexts and underscores the critical role of the self-system in the stress-disclosure relationship. The moderating process of hostility aligns with self-threat theory, namely, when a narcissist’s grandiose self conflicts with reality (e.g., others can overcome adversity and ultimately achieve success), the self perceives a sense of threat, thereby generating strong motivations to defend status, enhance image, or seek revenge [79–81]. This regulatory process of maintaining self-image [82] may manifest in this study as the hostility activated by the imperfect model. Specifically, when competitive narcissists participate in a sharing session, the high similarity between the imperfect role model and their own real-life experiences may lead to diminished self-esteem, thereby activating hostility to repair self-esteem. Furthermore, the study also revealed the complex role of horizontal comparison. Previous research has found that peer models, due to their similarity to individuals, are more likely to elicit benefits such as empathy. However, this study found that peer models do not necessarily always lead individuals to strive to improve themselves to narrow the gap with the model; they may also lead to attempts to weaken others’ advantages to reduce the gap. This may be because the imperfect model, being a peer who succeeded despite experiencing adversity, evokes envy. Research has found that people often envy advantaged others who are spatially close, of similar age, or have similar backgrounds. When individuals have more knowledge about the advantages of similar others, the induced social comparison prompts the generation of envy [83]. Individuals with competitive narcissism traits are highly sensitive to negative experiences and emotions [84] and are more prone to experiencing envy, a hostile social emotion, due to self-regulation difficulties [72,85]. This competitive narcissistic strategy essentially transfers self-threat through external attribution [68].

Ultimately, this study provides support for the diversity of meaning repair. The Meaning Maintenance Model posits that after meaning is disrupted, it can be repaired through assimilation, accommodation, affirmation, abstraction, and assembly. Among these, the affirmation strategy is a compensatory strategy, referring to individuals strengthening their already held meaning system and affirming its correctness more strongly after a meaning violation [86]. In this study, for individuals with competitive narcissistic traits, this meaning repair strategy manifested as activated hostility, thereby reinforcing their established cognitions regarding their own strengths and self-worth, and achieving meaning restoration as well as defense against threats. Unfortunately, even if individuals can recover from self-threat caused by comparison through hostility, research suggests that this defensive repair strategy often runs counter to the narcissistic individual’s goal of forming a grandiose self. This defensive motivation is highly correlated with more pathological vulnerability, higher impulsivity, and greater irritability [14,87]. Moreover, compared to the benign envy generated by admiring narcissism, which makes people more willing to exert effort for self-improvement, this malicious envy and hostility will trigger more frustration and avoidance behaviors [88].

### 5.2. Study 2: The chain mediating role of participation willingness on distress disclosure and the moderating role of adversity perception

Chain mediation analysis indicated that participation willingness influences distress disclosure by increasing stress and decreasing meaning in life. Bootstrap tests showed that the total indirect effect was significant (β = −0.20, 95% CI=[−0.32, −0.09]), while the direct effect was not significant (β = −0.17, 95% CI=[0.18, −0.42]), constituting a full mediation model. This suggests that participation willingness does not directly alter an individual’s tendency for distress disclosure but rather functions through the transformation process of psychological mechanisms. More critically, multi-group analysis revealed that this chain mechanism was significant only in the “adversity perception group.” In the prosperity perception group, the direct path was not significant (β = 0.15, 95% CI=[−0.32, −0.09]), and the effect size indicated that participation willingness inhibited distress disclosure. Cross-group comparison found a significant difference in the direct path of “participation willingness → distress disclosure” between the two groups (CR = −2.79). In the adversity perception group, the direct effect of “participation willingness → distress disclosure” was significant (β = −0.56, 95% CI=[−0.93, −0.22]), and the effect size indicated that participation willingness at this point promotes distress disclosure. This indicates that how individuals define others’ experiences (as prosperity or adversity) will alter the psychological response pathway. After being exposed to an imperfect model in an adverse context, individuals willing to participate in subsequent failure sharing sessions exhibited a greater tendency for distress disclosure compared to individuals exposed to a perfect model.

In summary, this seemingly paradoxical phenomenon can be profoundly explained from the perspective of Meaning Making Theory (MMT) [86]. According to MMT, every individual possesses a global meaning system that provides a cognitive framework for interpreting experiences and behavioral motivations. When individuals encounter events that challenge this framework, such as discovering their own shortcomings when comparing themselves with a role model, a meaning violation occurs. This cognitive discrepancy can cause stress or distress, thereby activating the individual’s meaning-making process [89]. In this study, an individual’s willingness to participate in others’ experience-sharing sessions can be understood as a psychological readiness to approach the role model and learn from new experiences by understanding others’ life stories, which may initiate social comparison or anticipatory anxiety about unknown experiences. Subsequently, this sense of stress can erode the individual’s clear perception of their own meaning in life (i.e., stress negatively predicts meaning), plunging the individual into temporary self-doubt or existential confusion. However, it is precisely this temporary lack of meaning or the need for reconstruction that ultimately promotes the individual’s tendency to disclose their own distress to others (i.e., meaning positively predicts distress disclosure). This pathway is highly consistent with the findings of Park and Baumeister, which guided participants to imagine future stressful events and found it increased their level of meaning-seeking [89]. Furthermore, research has found that disclosing negative emotions is a way to promote meaning-seeking [90], which also supports the findings of this study. In other words, disclosure is not merely emotional catharsis but also an active practice of meaning construction.

It is worth noting that an individual’s willingness to participate in failure sharing sessions may reflect their inherently higher disposition for disclosure. However, the results of the multi-group analysis showed that participation willingness significantly promoted subsequent distress disclosure only when the individual perceived others’ experiences as ‘adversity.’ This indicates that even if individuals possess a higher original tendency to disclose, the perfect model (prosperity perception) may still exert pressure from social comparison on them, thereby producing an inhibitory effect. This further supports the critical initiating role of the imperfect model in breaking down individuals’ psychological defenses and transforming the tendency to disclose into actual disclosure behavior.

The differential effects of prosperity and adversity perceptions are also traceable in previous research. Studies by Pierceall et al. indicate that 75% of college students perceive themselves as having moderate stress, while 12% experience high stress [91]. Students’ academic and employment stress may stem from excessively high expectations for achievement or overestimation of difficulty [92]. Perfect models may further elevate such expectations, thereby imposing stronger stress and inhibitory effects on disclosure on individuals. In contrast, imperfect models demonstrate the process of individuals overcoming adversity; because they are closer to individuals’ real-life experiences, they may encourage individuals to accept their current shortcomings and adjust unreasonable expectations. This process aligns with the meaning-focused coping process, where when individuals face an unattainable goal that cannot be achieved, they need to reformulate meaningful and realistic goals to alleviate distress and construct more adaptive meaning frameworks [93].

### 5.3. Limitations and practical implications

#### 5.3.1. Limitations.

First, the cross-sectional design makes it difficult to verify causal relationships between variables. Study 1 adopted a questionnaire survey method, and the relationships between variables were essentially correlational. Although the mediation model was constructed based on theory, the possibility of reverse causality or third variables cannot be completely ruled out. Future research could adopt longitudinal tracking designs or multi-time point measurements to more rigorously examine the dynamic causal relationships between variables.

Second, the sample was limited to the college student population, so caution is needed when generalizing the conclusions to high-pressure environments such as the workplace. Although college students face multiple pressures such as academic and employment stress, the nature of their stressors, social support systems, and meaning-making approaches may differ from those of the working population. In the workplace environment, factors such as performance pressure, organizational competition, and supervisor-subordinate relationships may have different effects on distress disclosure. Future research should extend to broader adult populations to test the cross-contextual stability of this study’s conclusions.

Third, the hostility is a branch within narcissism theory and cannot comprehensively explain the distinction between adaptive and non-adaptive responses in the meaning repair process. Admiring narcissism and competitive narcissism may differ in their meaning repair strategies; the former may be more likely to manifest as cognitive-level affirmation strategies, while the latter may involve behavioral-level belittling or aggression. Future research could adopt multi-method measurements or integrate more domains, such as benign envy and malicious envy, to further explore the different mechanisms at play in meaning repair.

Fourth, the uncontrolled gender variable may have potential effects on the research results. The proportion of female participants in this study was relatively high (females accounted for 77.8% in Study 2), and previous research indicates that there may be gender differences in distress disclosure, stress coping, and meaning-making approaches. Whether the effect of promoting disclosure in the adversity perception group in this study is equally significant in male populations requires further examination. Future research could consider gender-matched sampling designs or include gender as a moderating variable in the model.

Fifth, the potential influence of cultural background was not discussed. The sample of this study was all from Chinese college students, and Chinese culture is characterized by typical collectivism, emphasizing interpersonal harmony and face maintenance. Against this cultural backdrop, distress disclosure may face stronger social inhibition—individuals may choose to hide negative emotions due to concerns about “losing face” or “troubling others.” Whether the effect of promoting disclosure in the adversity perception group in Study 2 is stronger in Western cultural contexts that emphasize individual expression and personal growth, or weaker in East Asian cultural contexts with more pronounced shame orientation, warrants examination through cross-cultural comparative research. Furthermore, individuals’ interpretation of imperfect models in collectivist cultures may also be culturally specific: narratives in traditional Chinese culture such as “sleeping on brushwood and tasting gall” or “failure is the mother of success” may provide natural cultural resources for meaning reconstruction in adversity, which might partially explain the positive effects observed in Study 2.

Sixth, social desirability effects may have potential influence. In Study 2, participation willingness was much higher than non-participation willingness, which may have been influenced by social desirability. Future research could incorporate behavioral indicators (such as actual participation rates, disclosure duration) or other-report data to more objectively measure disclosure behavior.

Seventh, the potential confounding effect of self-selection bias. This study found that individuals willing to participate in “failure sharing sessions” exhibited a stronger tendency for disclosure, but it cannot be ruled out that this group inherently possesses higher trait disclosure. This “spontaneous tendency” may be intertwined with experimentally induced “state willingness.” Although the multi-group comparisons indicated to some extent the moderating role of situational perception, future research should measure participants’ baseline disclosure levels before the experiment, or adopt mandatory situation presentation designs, to more purely isolate the inducing effect of imperfect models on distress disclosure.

#### 5.3.2. Theoretical contributions and practical implications.

At the theoretical level, this study, by introducing the Meaning Maintenance Model (MMM), successfully integrated the paradoxical findings of Study 1 and Study 2, revealing the dual pathways of the imperfect model effect and its critical boundary conditions. Study 1 indicated that when individuals are exposed to an imperfect model, meaning violation primarily triggers defensive meaning repair (such as the affirmation strategy in individuals with high hostility), leading to the inhibition of disclosure. Study 2 showed that when individuals are primed by an imperfect model, the meaning violation caused by this experience may imply that the individual has accepted the possibility of succeeding despite imperfection, thereby actively adjusting goals and seeking support. Consequently, the anticipation of participating in more subsequent sharing sessions oriented them towards constructive meaning reconstruction, which in turn facilitated disclosure. This integrative framework extends the Meaning Maintenance Model from a mere theory of meaning repair to a unified theoretical model explaining social comparison and disclosure behavior.

At the practical level, this study offers multifaceted implications for mental health interventions in colleges and universities: First, embed imperfect model cases in group counseling, presenting the complete narrative of the model’s “failure-reflection-growth” rather than merely showcasing their successful outcomes. Through parallel social comparison (as opposed to upward comparison), help individuals recognize the universality and transform ability of setbacks, thereby reducing the shame associated with disclosure. Second, design differentiated intervention strategies for individuals with narcissistic traits, especially those with high hostility, to help them learn more adaptive meaning repair strategies and reduce potential psychological and behavioral issues such as aggression. Third, leverage meaning in life interventions to promote adaptive disclosure. Guide individuals to reframe failure experiences as meaningful turning points in their life stories, transforming distress disclosure from “emotional catharsis” into a “narrative tool for self-growth” [78].

Future research could attempt to conduct longitudinal tracking studies to examine the dynamic changes in meaning repair strategies and their long-term impact on mental health. Secondly, extend the research to multicultural contexts to test the potential inhibitory effect of shame-oriented collectivist cultures on the meaning reconstruction pathway. Thirdly, adopt multi-method measurements (such as physiological indicators, behavioral observations) to verify the reliability of self-report results. Finally, develop intervention programs based on the Meaning Maintenance Model and test their effectiveness in promoting mental health through randomized controlled trials.

## Supporting information

S1 Dataset Data for Study1. (XLSX)

S2 Dataset Data for Study2. (XLSX)

S3 File Detailed results of the multi-group structural equation models conducted in IBM SPSS Amos, including model fit indices, parameter estimates, and bootstrap confidence intervals for indirect effects. (PDF)
