# Supplementary material for: From defense to reconstruction: The hostility-meaning dual-path model of how observing others’ adversity influences distress disclosure
Source: PLoS One. 2026 Jun 8;21(6):e0350109. doi: 10.1371/journal.pone.0350109 (PMC13245792; doi:10.1371/journal.pone.0350109)
Supplement: S3 File — (PDF) [file pone.0350109.s003.pdf]

Analysis Summary

- **Software**: IBM SPSS Amos (version not specified in output)
- **Estimation Method**: Maximum Likelihood (ML)
- **Bootstrap**: 2000 resamples (Bias-corrected percentile method, 90% confidence intervals)
- **Groups**:
  - Prosperity group: N = 303
  - Adversity group: N = 233
- **Model Type**: Recursive multi-group model

1. Path Diagrams

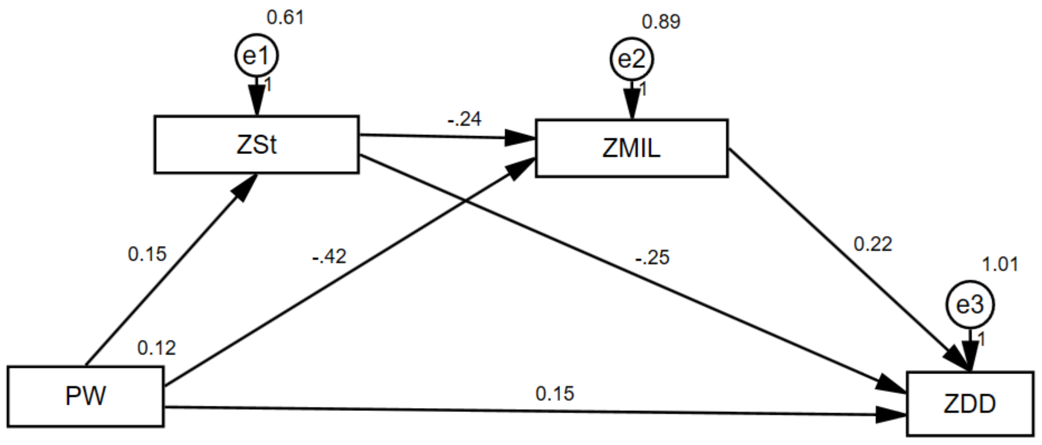

Path diagram for the Prosperity group (standardized estimates)

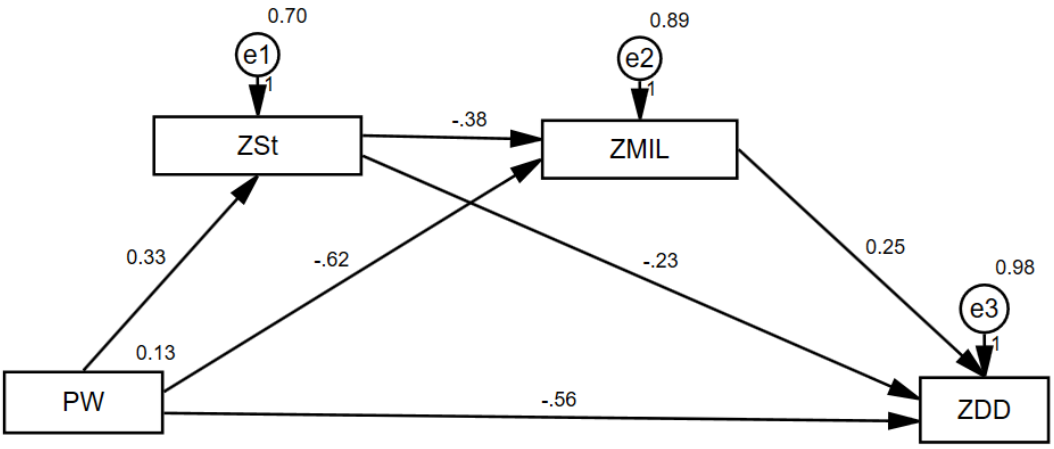

Path diagram for the Adversity group (standardized estimates)

2. Model Fit Indices

Table S6-1. Model Fit Summary

Model	NPAR	CMIN	DF	P	CMIN/DF	CFI	TLI	GFI
Unconstrained	20	0.000	0	-	-	1.000	1.000	1.000
Structural weights	14	12.089	6	0.060	2.015	0.953	0.905	0.989
Structural covariances	13	12.383	7	0.089	1.769	0.958	0.928	0.989
Structural residuals	10	13.695	10	0.187	1.369	0.971	0.966	0.987

Note: The unconstrained model was used for parameter estimation. Nested models were tested for cross-group comparisons.

3. Parameter Estimates

Table S6-2. Unstandardized Regression Weights (Maximum Likelihood Estimates)

Path	Prosperity Estimate (SE)	Prosperity CR	Prosperity p	Adversity Estimate (SE)	Adversity CR	Adversity p
ZSt ← PW	0.154 (0.130)	1.188	0.235	0.331 (0.153)	2.161	0.031
ZMIL ← ZSt	-0.243 (0.070)	-3.481	<0.001	-0.382 (0.074)	-5.154	<0.001
ZMIL	-0.423	-2.687	0.007	-0.616	-3.524	<0.001

← PW	(0.158)			(0.175)		
ZDD	0.218	3.556	<0.001	0.246	3.558	<0.001
←	(0.061)			(0.069)		
ZMIL						
ZDD	-0.248	-3.276	0.001	-0.232	-2.822	0.005
← ZSt	(0.076)			(0.082)		
ZDD	0.147	0.868	0.385	-0.561	-2.973	0.003
← PW	(0.170)			(0.189)		

Table S6-3. Standardized Regression Weights

Path	Prosperity	Adversity
ZSt ← PW	0.068	0.140
ZMIL ← ZSt	-0.194	-0.313
ZMIL ← PW	-0.150	-0.214
ZDD ← ZMIL	0.202	0.231
ZDD ← ZSt	-0.184	-0.179
ZDD ← PW	0.048	-0.183

Table S6-4. Squared Multiple Correlations (R^2)

Variable	Prosperity	Adversity
ZSt	0.005	0.020
ZMIL	0.064	0.162
ZDD	0.088	0.179

4. Indirect Effects (Bootstrap 90% Bias-Corrected CI)

Table S6-5. User-defined Estimands (Indirect Effects)

Estimand	Definition	Estimate	Lower	Upper	p
int1	a1 × a5 (PW→ZSt→ZDD)	-0.038	-0.119	0.011	0.205
Int2	a4 × a3 (PW→ZMIL→ZDD)	-0.092	-0.184	-0.033	0.004
int3	a1 × a2 × a3 (PW→ZSt→ZMIL→ZDD)	-0.008	-0.031	0.001	0.131
inttol	Total indirect (prosperity)	-0.139	-0.247	-0.043	0.014
int1b	b1 × b5 (PW→ZSt→ZDD)	-0.126	-0.256	-0.028	0.028
int2b	b4 × b3 (PW→ZMIL→ZDD)	-0.151	-0.337	-0.049	0.006
int3b	b1 × b2 × b3 (PW→ZSt→ZMIL→ZDD)	-0.031	-0.081	-0.007	0.016
inttolb	Total indirect (adversity)	-0.309	-0.556	-0.123	0.004

Note: Confidence intervals and p-values are based on 2000 bootstrap samples using bias-corrected percentile method.
