# Supplementary material for: From defense to reconstruction: The hostility-meaning dual-path model of how observing others’ adversity influences distress disclosure
Source: PLoS One. 2026 Jun 8;21(6):e0350109. doi: 10.1371/journal.pone.0350109 (PMC13245792; doi:10.1371/journal.pone.0350109)
Supplement: Attachment — Submitted filename: Response to Reviewers.docx [file pone.0350109.s004.docx]

Dear Editors and Reviewers:

Thank you for your letter and for the reviewers’ comments concerning our manuscript entitled “From defense to reconstruction: The hostility-meaning dual-path model of how observing others' adversity influences self-negative disclosure” (PONE-D-25-31226).Those comments are all valuable and very helpful for revising and improving our paper, as well as the important guiding significance to our researches. We have studied comments carefully and have made correction which we hope meet with approval. Revised portion are marked in red in the paper. The main corrections in the paper and the responds to the reviewer’s comments are as flowing:

**To Editor**

**Comment 1**

Please ensure that your manuscript meets PLOS ONE's style requirements, including those for file naming.

**Response**

Thank you for your reminder. We have carefully revised the manuscript to ensure that it fully complies with PLOS ONE’s style requirements, including the required file naming conventions.

**Comment 2**

We note that the grant information you provided in the ‘Funding Information’ and ‘Financial Disclosure’ sections do not match. When you resubmit, please ensure that you provide the correct grant numbers for the awards you received for your study in the ‘Funding Information’ section.

**Response**

Thank you for pointing this out. We have revised the Funding Statement and updated it with the correct grant numbers. The information provided in the “Funding Information” and “Financial Disclosure” sections is now consistent.

**Comment 3**

Thank you for stating the following financial disclosure: General Project of the 2020 Annual Scientific Research Open Fund of the Research Center for College Student Development and Innovation Education - Key Research Base of Humanities and Social Sciences in Hubei Provincial Colleges and Universities. Please state what role the funders took in the study. If the funders had no role, please state: "The funders had no role in study design, data collection and analysis, decision to publish, or preparation of the manuscript." If this statement is not correct you must amend it as needed. Please include this amended Role of Funder statement in your cover letter; we will change the online submission form on your behalf.

**Response**

We thank the reviewer for this reminder. We have revised the funding information and clarified the role of the funders. The updated **Funding Statement** and the **Role of the Funder** have been included in the **cover letter**, as requested. The revised statement reads as follows:

*“****Funding Statement***

*This research was supported by the 2022 Project of the Center for Innovation and Development of Ideological and Political Work in Higher Education Institutions of the Ministry of Education (Wuhan Donghu University), under Grant No. WHDHSZZX2022003. The funders had no role in study design, data collection and analysis, decision to publish, or preparation of the manuscript.”*

**Comment 4**

Please include captions for your Supporting Information files at the end of your manuscript, and update any in-text citations to match accordingly.

**Response**

Thank you for the suggestion. We have added captions for all Supporting Information files at the end of the manuscript. In addition, the file names used in the manuscript have been revised to match the corresponding Supporting Information files. We also clarified which figures correspond to the results of each study.

**Comment 5**

Thank you for providing your underlying data as Supporting Information. We note that the data set contains text or data that is not in English. Please note that PLOS is an English-language publisher, so we require data sets to be provided in English as well. Please upload an English-language version of your data set.

**Response**

Thank you for your comment. We have translated the dataset into English and uploaded an English-language version as Supporting Information in accordance with PLOS ONE’s requirements.

**Comment 6**

We are unable to open your Supporting Information file [renamed_b7bb3.amw]. Please kindly revise as necessary and re-upload.

**Response**

Thank you for informing us. The file [renamed_b7bb3.amw] is an AMOS model file. We have modified the file names of the images used in the manuscript in accordance with the formatting requirements, corresponded to these images in the Supporting Information section of the manuscript using the same file names, and provided explanations of what each image represents in the study.

**Comment 7**

If the reviewer comments include a recommendation to cite specific previously published works, please review and evaluate these publications to determine whether they are relevant and should be cited. There is no requirement to cite these works unless the editor has indicated otherwise.

**Response**

Thank you for the reminder. We carefully reviewed the reviewer comments and noted that no specific recommendations for additional citations were provided.

**Comment 8**

Please ensure that you refer to Table 9 in your text as, if accepted, production will need this reference to link the reader to the Table.
**Response**

Thank you for the reminder. We have revised the manuscript and added the corresponding in-text citation to **Table 9** to ensure that the table is properly referenced in the text.

**Comment 9**

We note that your author list was updated during the revision process. In order to add or remove authors or update the order of the author byline after initial submission, we ask that authors complete an Authorship Change Request form. You may review our full authorship change policy and download the Authorship Change Request form here: https://journals.plos.org/plosone/s/authorship#loc-authorship-changes.

**Response**

Thank you for the reminder. We have completed the **Authorship Change Request form** in accordance with the journal’s policy and submitted it along with the revised manuscript.

**To reviewer 1**

**1. Introduction**

**Comment 1**

The theories mentioned will need to be combined to demonstrate how they interact. It is recommended to narrow the theoretical scope by emphasising the most central mechanism.

**Response**

We sincerely thank the reviewer for this suggestion. We have streamlined the theoretical framework by positioning the Meaning **Maintenance Model (MMM)** as the primary overarching theory. In the revised Introduction and Discussion, we now explicitly articulate how the MMM integrates social comparison and self-defense mechanisms. Specifically, we clarify the interaction between "meaning violation" (caused by social comparison) and the subsequent paths of defensive compensation (Study 1) and constructive structural realignment (Study 2). This refinement ensures a more focused and cohesive explanation of the core psychological mechanisms at play.

**Comment 2**

Some key constructs, such as imperfect role models, hostility, and meaning in life, lack operational precision. Provide clear, operational definitions for each construct.

**Response**

We appreciate this important suggestion. In the revised manuscript, we added **explicit operational definitions** for the core constructs. Specifically, *imperfect role models* are defined as successful individuals whose narratives explicitly include experiences of failure or struggle prior to achievement. *Hostility* is defined as a stable individual difference reflecting a tendency to perceive interpersonal situations as competitive or threatening and to respond with defensive antagonism. *Meaning in life* is defined as individuals’ perceived sense of purpose, coherence, and significance in their lives. These definitions have been incorporated into the Introduction and Methods sections to clarify the conceptual and empirical scope of each construct.

**Comment 3**

Strengthen the empirical rationale by explicitly citing inconsistencies or gaps in prior studies.

**Response**

Thank you for this valuable suggestion. In response, we expanded the literature review to more clearly highlight existing gaps and inconsistencies in prior research. Specifically, we emphasize three unresolved issues: (1) prior studies have primarily focused on the negative consequences of upward social comparison, leaving its potential constructive effects underexplored; (2) empirical research on imperfect role models remains limited compared to the extensive literature on idealized successful figures; and (3) previous work rarely integrates defensive responses and meaning reconstruction within a unified theoretical framework. By addressing these gaps, the revised manuscript clarifies the theoretical and empirical motivation for the present research.

**2. Methodology**

**Comment 4**

Sample homogeneity: predominantly female university students limit external validity. It should be discussed in the Discussion Section (Gender Differences) and also included in the Limitations Section.

**Response**

We sincerely appreciate the reviewer’s suggestion to address the potential influence of uncontrolled variables. In the revised manuscript, we have added a dedicated discussion in the Limitations section to acknowledge these factors. Specifically, we analyzed how variables such as gender distribution (notably the higher proportion of female participants), cultural background, and social desirability bias may have influenced participants' self-reporting of distress and meaning in life. We have also suggested that future studies could employ more diverse samples and control for social desirability to further validate the generalizability of our findings. This addition provides a more comprehensive and balanced perspective on the scope of our results.

**Comment 5**

Self-selection bias: participants who attend “failure-sharing sessions” may already favour disclosure. It should be discussed in the Discussion section (Gender Differences) and also included in the limitations section.

**Response**

We sincerely thank the reviewer for pointing out this critical methodological issue. We agree that participants’ initial willingness to engage with failure-related content may be confounded by their trait-level predisposition toward self-disclosure (self-selection bias). It is also important to clarify that in the present study, **participation willingness refers to individuals’ willingness to attend a subsequent experience-sharing session**, rather than indicating that they would necessarily disclose their own experiences during the session.

To address this concern, we have implemented the following revisions:

**In the Discussion (Section 5.2):** We have added an analysis exploring whether "participation willingness" may partially reflect pre-existing disclosure tendencies. We argue that while such a tendency might exist, the significant difference between the prosperity-perception group and the adversity-perception group suggests that the situational interpretation (imperfect role model) still plays a crucial moderating role beyond individual traits.

**In the Limitations (Section 5.4.1):** We have added a new limitation (the seventh point) specifically addressing this potential self-selection bias. We acknowledge that future research should measure baseline disclosure tendencies or adopt a forced-exposure design to better isolate the causal effect of the experimental manipulation.

**Comment 6**

Lack of manipulation checks for scenario validity in Study 2.

**Response**

We sincerely appreciate the reviewer’s suggestion to ensure the internal validity of our experimental manipulation. In the revised manuscript, we have now included a detailed **manipulation check** in Section 3.2.2. To verify the effectiveness of the scenarios (Failure vs. Success), we performed a Chi-square test between the experimental groups and participants' subjective perceptions (Adversity vs. Prosperity). The results indicated a highly significant association, χ²(1, N = 536) = 312.87, p < .001, φ = 0.76, with 89.2% of the failure group perceiving the scenario as adversity and 87.5% of the success group perceiving it as prosperity. Furthermore, an additional Chi-square analysis confirmed that the experimental manipulation did not significantly impact participation willingness, χ²(1, N = 536) = 0.14，p = 0.712，φ = -0.016, thereby ruling out participation intent as a confounding variable. These results provide a robust foundation for our subsequent mediation analyses.

**Comment 7**

The Psychometric properties in brief of the original measures, as well as the translated versions, will be needed to add to each measure. Additionally, some items from each measure are preferred to be included in each measure.

**Response**

We sincerely appreciate the reviewer's constructive suggestion. In the revised manuscript (Sections 3.1.2 and 3.2.3), we have comprehensively updated the descriptions for all measurement scales. Specifically, we have added:

Psychometric Properties: We now report the Cronbach’s α coefficients for the original English versions, the previously validated Chinese versions, and the current study's internal consistency (including sub-dimension reliability where applicable).

Sample Items: Specific example items have been included for each scale to enhance clarity.

**Comment 8**

Ethical consideration: no debriefing procedures (participants' support) mentioned after inducing failure scenarios. This needs to be mentioned in the Methodology Section.

**Response**

We thank the reviewer for this insightful suggestion. In the revised manuscript, we have added a detailed description of the debriefing procedure in Section 3.2.2. Specifically, we clarified the experimental nature of the failure scenarios to the participants and provided a multi-layered support system, including immediate on-site psychological guidance and contact information for the university’s counseling services.

**3. Results**

**Comment 9**

Clarify in the response letter how PROCESS was used to assess the CFA.

**Response**

We are grateful for the opportunity to clarify our statistical approach. We would like to clarify that PROCESS (Hayes, 2022) was utilized exclusively for testing our mediation, serial mediation, and moderated mediation hypotheses (Models 4, 6, and 7), rather than for performing Confirmatory Factor Analysis (CFA). In our data analysis workflow, we focused on path analysis and the estimation of indirect effects using the bias-corrected percentile Bootstrap method. We have carefully audited our manuscript and removed any ambiguous phrasing that might have suggested the use of PROCESS for CFA. Instead, we have explicitly stated that PROCESS was employed to evaluate the proposed theoretical model and the significance of the paths between variables. This clarification ensures that our statistical reporting aligns strictly with the functions of the software used

**4. Discussion**

**Comment 10**

Temper causal language (use “may” or “suggests” instead of “reveals” or “proves”).

**Response**

We sincerely thank the reviewer for this valuable guidance on academic precision. We have carefully audited the entire manuscript, particularly the Discussion section, to refine our phrasing. To address this concern, we have replaced absolute causal terms such as "reveals," "proves," or "determines" with more appropriate, nuanced expressions.

**Comment 11**

Acknowledge and discuss uncontrolled variables (e.g., gender, culture, social desirability).

**Response**

We sincerely appreciate the reviewer’s suggestion to address the potential influence of uncontrolled variables. In the revised manuscript, we have added a dedicated discussion in the Limitations section to acknowledge these factors. Specifically, we analyzed how variables such as gender distribution (notably the higher proportion of female participants), cultural background, and social desirability bias may have influenced participants' self-reporting of distress and meaning in life. We have also suggested that future studies could employ more diverse samples and control for social desirability to further validate the generalizability of our findings. This addition provides a more comprehensive and balanced perspective on the scope of our results.

**Comment 12**

Distinguish empirical results from theoretical interpretation explicitly.

**Response**

We thank the reviewer for this insightful feedback on the clarity of our presentation. We have carefully restructured the Discussion section to clearly distinguish between direct empirical findings and our theoretical interpretations. Specifically, we have revised the language to ensure that empirical evidence is presented first as the factual basis, followed by transitional phrases that explicitly identify subsequent theoretical derivations. This structural change ensures a more transparent logical flow and prevents any potential confusion between data and interpretation.

**To Reviewer 2**

**Comment 13**

About statistical anaysis, the author shows results in tables using mean scores, which seems that the mean showed in tables results of data placed above or under theorical median (zero), but this is a statistical error due to numerical data (scores) were transform to cathegorical data, therefore, this data, which does not follow a normal distribution and standar desviation is a property of numerical data normally distributed.Furthermore, authors must explain if Pearson's correlation analysis used with this cathegorical data too.

**Response**

We thank the reviewer for this helpful comment and would like to clarify a possible misunderstanding regarding the statistical presentation.

Regarding the mean values reported in the tables, all continuous variables (Stress, Self-esteem, Distress Disclosure, and Hostility) were **standardized prior to analysis**, and thus **all data presented in the tables are derived from these Z-standardized values**. As a result, the means appear close to zero by definition. Importantly, **the continuous variables were not transformed into categorical variables**; they remained continuous variables throughout the analyses. Regarding the categorical variable **Participation (0/1)**, it was used only as a grouping variable in the mediation analysis and was **not included in the descriptive statistics or correlation matrix**.

Finally, **Pearson correlation analyses were conducted only among the continuous variables**. The binary variable (Participation) was analyzed using the **PROCESS macro for path analysis**, which is appropriate for models including categorical predictors.

We have clarified these points in the revised manuscript to ensure that the distinction between standardized continuous variables and categorical variables is clearly explained.

**Comment 14**

RS in table 5 is not defined.

**Response**

We sincerely apologize for this clerical error. The term "RS" was a typographical error and should have been written as "SE," representing the Standard Error of the effect estimates. We have corrected this abbreviation to "SE" in Table 5 and throughout the revised manuscript to ensure consistency and clarity. We appreciate the reviewer’s meticulousness in identifying this oversight.

**Comment 15**

Lack empirical evidence for hypotesis 5.

**Response**

Regarding the empirical evidence for Hypothesis 5, we have significantly strengthened the analysis in Section 4.4.4. First, we provided a detailed comparison of the model structures, identifying a transition from a full mediation model in the prosperity-perception group to a partial mediation model in the adversity-perception group. Second, we utilized the Critical Ratio (CR) method to statistically confirm that the direct path from participation willingness to distress disclosure differs significantly across groups (CR = -2.81, p < .01). Finally, we added a bootstrap-based indirect effect comparison, showing that the chain mediation mechanism is specifically activated and strengthened when participants perceive the scenario as adversity. These statistical findings collectively provide robust empirical support for the moderating role of situational perception as proposed in Hypothesis 5.

**Comment 16**

Point 5.2 about complementarity of hostility and meaning reconstruction only have one comparative reference, this is insufficient for discuss the topic, author must present more references about it.

**Response**

We sincerely appreciate the reviewer's suggestion to strengthen the theoretical foundation of this section. In the revised manuscript, we have expanded the discussion in Section 5.2 by incorporating multiple additional references that explore the relationship between hostility (rivalry/admiration) and meaning-making processes. These new citations provide a more robust comparative framework, further validating our argument regarding how hostility levels interact with the reconstruction of life meaning in the face of "imperfect role models." We believe these additions significantly enhance the depth and academic rigor of our discussion

Once again, we sincerely thank the editor and reviewers for their constructive feedback. We believe that these revisions have substantially improved the clarity, rigor, and contribution of the manuscript.

Sincerely,
The Authors
