# Supplementary material for: From defense to reconstruction: The hostility-meaning dual-path model of how observing others’ adversity influences distress disclosure
Source: PLoS One. 2026 Jun 8;21(6):e0350109. doi: 10.1371/journal.pone.0350109 (PMC13245792; doi:10.1371/journal.pone.0350109)
Supplement: Peer review history

# Peer review: Decision Letter 0

**DOI:** 10.1371/journal.pone.0350109.r001

**Authors:** Atreyee Bhattacharyya

20 Jan 2026

PONE-D-25-31226

From defense to reconstruction: The hostility-meaning dual-path model of how observing others’ adversity influences self-negative disclosure

PLOS One

Dear Dr. Wang,

Thank you for submitting your manuscript to PLOS ONE. After careful consideration, we feel that it has merit but does not fully meet PLOS ONE’s publication criteria as it currently stands. Therefore, we invite you to submit a revised version of the manuscript that addresses the points raised during the review process.

Please submit your revised manuscript by Mar 06 2026 11:59PM. If you will need more time than this to complete your revisions, please reply to this message or contact the journal office at plosone@plos.org. When you’re ready to submit your revision, log on to https://www.editorialmanager.com/pone/ and select the ’Submissions Needing Revision’ folder to locate your manuscript file.

Please include the following items when submitting your revised manuscript:

- A letter that responds to each point raised by the academic editor and reviewer(s). You should upload this letter as a separate file labeled ’Response to Reviewers’.
- A marked-up copy of your manuscript that highlights changes made to the original version. You should upload this as a separate file labeled ’Revised Manuscript with Track Changes’.
- An unmarked version of your revised paper without tracked changes. You should upload this as a separate file labeled ’Manuscript’.

If you would like to make changes to your financial disclosure, please include your updated statement in your cover letter. Guidelines for resubmitting your figure files are available below the reviewer comments at the end of this letter.

If applicable, we recommend that you deposit your laboratory protocols in protocols.io to enhance the reproducibility of your results. Protocols.io assigns your protocol its own identifier (DOI) so that it can be cited independently in the future. For instructions see: https://journals.plos.org/plosone/s/submission-guidelines#loc-laboratory-protocols. Additionally, PLOS ONE offers an option for publishing peer-reviewed Lab Protocol articles, which describe protocols hosted on protocols.io. Read more information on sharing protocols at https://plos.org/protocols?utm_medium=editorial-email&utm_source=authorletters&utm_campaign=protocols.

We look forward to receiving your revised manuscript.

Kind regards,

Atreyee Bhattacharyya, Ph.D.

Guest Editor

PLOS One

Journal Requirements:

When submitting your revision, we need you to address these additional requirements.

1. Please ensure that your manuscript meets PLOS ONE’s style requirements, including those for file naming. The PLOS ONE style templates can be found at

https://journals.plos.org/plosone/s/file?id=wjVg/PLOSOne_formatting_sample_main_body.pdf and

https://journals.plos.org/plosone/s/file?id=ba62/PLOSOne_formatting_sample_title_authors_affiliations.pdf

2. We note that the grant information you provided in the ‘Funding Information’ and ‘Financial Disclosure’ sections do not match.

When you resubmit, please ensure that you provide the correct grant numbers for the awards you received for your study in the ‘Funding Information’ section.

3. Thank you for stating the following financial disclosure:

General Project of the 2020 Annual Scientific Research Open Fund of the Research Center for College Student Development and Innovation Education - Key Research Base of Humanities and Social Sciences in Hubei Provincial Colleges and Universities

Please state what role the funders took in the study. If the funders had no role, please state: "The funders had no role in study design, data collection and analysis, decision to publish, or preparation of the manuscript."

If this statement is not correct you must amend it as needed.

Please include this amended Role of Funder statement in your cover letter; we will change the online submission form on your behalf.

4. Please include captions for your Supporting Information files at the end of your manuscript, and update any in-text citations to match accordingly. Please see our Supporting Information guidelines for more information: http://journals.plos.org/plosone/s/supporting-information.

5. Thank you for providing your underlying data as Supporting Information.

We note that the data set contains text or data that is not in English. Please note that PLOS is an English-language publisher, so we require data sets to be provided in English as well. Please upload an English-language version of your data set.

This will also allow us to determine if your data follows PLOS standards per our Data Availability policy here: https://journals.plos.org/plosone/s/data-availability

6. We are unable to open your Supporting Information file [renamed_b7bb3.amw]. Please kindly revise as necessary and re-upload.

7. If the reviewer comments include a recommendation to cite specific previously published works, please review and evaluate these publications to determine whether they are relevant and should be cited. There is no requirement to cite these works unless the editor has indicated otherwise.

Reviewers’ comments:

Reviewer’s Responses to Questions

**Comments to the Author**

1. Is the manuscript technically sound, and do the data support the conclusions?

The manuscript must describe a technically sound piece of scientific research with data that supports the conclusions. Experiments must have been conducted rigorously, with appropriate controls, replication, and sample sizes. The conclusions must be drawn appropriately based on the data presented.

Reviewer #1: Yes

Reviewer #2: Partly

**********

2. Has the statistical analysis been performed appropriately and rigorously?

Reviewer #1: N/A

Reviewer #2: No

**********

3. Have the authors made all data underlying the findings in their manuscript fully available?

The PLOS Data policy requires authors to make all data underlying the findings described in their manuscript fully available without restriction, with rare exception (please refer to the Data Availability Statement in the manuscript PDF file). The data should be provided as part of the manuscript or its supporting information, or deposited to a public repository. For example, in addition to summary statistics, the data points behind means, medians and variance measures should be available. If there are restrictions on publicly sharing data—e.g. participant privacy or use of data from a third party—those must be specified.

Reviewer #1: Yes

Reviewer #2: Yes

**********

4. Is the manuscript presented in an intelligible fashion and written in standard English?

PLOS ONE does not copyedit accepted manuscripts, so the language in submitted articles must be clear, correct, and unambiguous. Any typographical or grammatical errors should be corrected at revision, so please note any specific errors here.

Reviewer #1: Yes

Reviewer #2: Yes

**********

5. Review Comments to the Author

Please use the space provided to explain your answers to the questions above. You may also include additional comments for the author, including concerns about dual publication, research ethics, or publication ethics. (Please upload your review as an attachment if it exceeds 20,000 characters)

Reviewer #1: In general, this is a well-written paper. The topic is a timely and meaningful research problem.

1-Introduction

1. The theories mentioned will need to be combined to demonstrate how they interact. It is recommended to narrow the theoretical scope by emphasising the most central mechanism.

2. Some key constructs, such as imperfect role models, hostility, and meaning in life, lack operational precision. Provide clear, operational definitions for each construct.

3. Strengthen the empirical rationale by explicitly citing inconsistencies or gaps in prior studies.

2. Methodology

4-Sample homogeneity: predominantly female university students limit external validity. It should be discussed in the Discussion Section (Gender Differences) and also included in the Limitations Section.

5-Self-selection bias: participants who attend “failure-sharing sessions” may already favour disclosure. It should be discussed in the Discussion section (Gender Differences) and also included in the limitations section.

6-Lack of manipulation checks for scenario validity in Study 2.

7-The Psychometric properties in brief of the original measures, as well as the translated versions, will be needed to add to each measure. Additionally, some items from each measure are preferred to be included in each measure.

8-Ethical consideration: no debriefing procedures (participants’ support) mentioned after inducing failure scenarios. This needs to be mentioned in the Methodology Section.

3. Results

9-Clarify in the response letter how PROCESS was used to assess the CFA.

4. Discussion

10-Temper causal language (use “may” or “suggests” instead of “reveals” or “proves”).

11. Acknowledge and discuss uncontrolled variables (e.g., gender, culture, social desirability).

12-Distinguish empirical results from theoretical interpretation explicitly.

Reviewer #2: About statistical anaysis, the author shows results in tables using mean scores, which seems that the mean showed in tables results of data placed above or under theorical median (zero), but this is a statistical error due to numerical data (scores) were transform to cathegorical data, therefore, this data, which does not follow a normal distribution and standar desviation is a property of numerical data normally distributed. Furthermore, authors must explain if Pearson’s correlation analysis used with this cathegorical data too. RS in table 5 is not defined. Lack empirical evidence for hypotesis 5; Point 5.2 about complementarity of hostility and meaning reconstruction only have one comparative reference, this is insufficient for discuss the topic, author must present more references about it.

**********

6. PLOS authors have the option to publish the peer review history of their article (what does this mean?). If published, this will include your full peer review and any attached files.

If you choose “no”, your identity will remain anonymous but your review may still be made public.

**Do you want your identity to be public for this peer review?** For information about this choice, including consent withdrawal, please see our Privacy Policy.

Reviewer #1: No

Reviewer #2: No

**********

[NOTE: If reviewer comments were submitted as an attachment file, they will be attached to this email and accessible via the submission site. Please log into your account, locate the manuscript record, and check for the action link "View Attachments". If this link does not appear, there are no attachment files.]

To ensure your figures meet our technical requirements, please review our figure guidelines: https://journals.plos.org/plosone/s/figures

You may also use PLOS’s free figure tool, NAAS, to help you prepare publication quality figures: https://journals.plos.org/plosone/s/figures#loc-tools-for-figure-preparation.

NAAS will assess whether your figures meet our technical requirements by comparing each figure against our figure specifications.

# Author comment: Author response to Decision Letter 1

**DOI:** 10.1371/journal.pone.0350109.r002

16 Apr 2026

Dear Editors and Reviewers:

Thank you for your letter and for the reviewers’ comments concerning our manuscript entitled “From defense to reconstruction: The hostility-meaning dual-path model of how observing others’ adversity influences self-negative disclosure” (PONE-D-25-31226).Those comments are all valuable and very helpful for revising and improving our paper, as well as the important guiding significance to our researches. We have studied comments carefully and have made correction which we hope meet with approval. Revised portion are marked in red in the paper. The main corrections in the paper and the responds to the reviewer’s comments are as flowing:

To Editor

Comment 1

Please ensure that your manuscript meets PLOS ONE’s style requirements, including those for file naming.

Response

Thank you for your reminder. We have carefully revised the manuscript to ensure that it fully complies with PLOS ONE’s style requirements, including the required file naming conventions.

Comment 2

We note that the grant information you provided in the ‘Funding Information’ and ‘Financial Disclosure’ sections do not match. When you resubmit, please ensure that you provide the correct grant numbers for the awards you received for your study in the ‘Funding Information’ section.

Response

Thank you for pointing this out. We have revised the Funding Statement and updated it with the correct grant numbers. The information provided in the “Funding Information” and “Financial Disclosure” sections is now consistent.

Comment 3

Thank you for stating the following financial disclosure: General Project of the 2020 Annual Scientific Research Open Fund of the Research Center for College Student Development and Innovation Education - Key Research Base of Humanities and Social Sciences in Hubei Provincial Colleges and Universities. Please state what role the funders took in the study. If the funders had no role, please state: "The funders had no role in study design, data collection and analysis, decision to publish, or preparation of the manuscript." If this statement is not correct you must amend it as needed. Please include this amended Role of Funder statement in your cover letter; we will change the online submission form on your behalf.

Response

We thank the reviewer for this reminder. We have revised the funding information and clarified the role of the funders. The updated Funding Statement and the Role of the Funder have been included in the cover letter, as requested. The revised statement reads as follows:

“Funding Statement

This research was supported by the 2022 Project of the Center for Innovation and Development of Ideological and Political Work in Higher Education Institutions of the Ministry of Education (Wuhan Donghu University), under Grant No. WHDHSZZX2022003. The funders had no role in study design, data collection and analysis, decision to publish, or preparation of the manuscript.”

Comment 4

Please include captions for your Supporting Information files at the end of your manuscript, and update any in-text citations to match accordingly.

Response

Thank you for the suggestion. We have added captions for all Supporting Information files at the end of the manuscript. In addition, the file names used in the manuscript have been revised to match the corresponding Supporting Information files. We also clarified which figures correspond to the results of each study.

Comment 5

Thank you for providing your underlying data as Supporting Information. We note that the data set contains text or data that is not in English. Please note that PLOS is an English-language publisher, so we require data sets to be provided in English as well. Please upload an English-language version of your data set.

Response

Thank you for your comment. We have translated the dataset into English and uploaded an English-language version as Supporting Information in accordance with PLOS ONE’s requirements.

Comment 6

We are unable to open your Supporting Information file [renamed_b7bb3.amw]. Please kindly revise as necessary and re-upload.

Response

Thank you for informing us. The file [renamed_b7bb3.amw] is an AMOS model file. We have modified the file names of the images used in the manuscript in accordance with the formatting requirements, corresponded to these images in the Supporting Information section of the manuscript using the same file names, and provided explanations of what each image represents in the study.

Comment 7

If the reviewer comments include a recommendation to cite specific previously published works, please review and evaluate these publications to determine whether they are relevant and should be cited. There is no requirement to cite these works unless the editor has indicated otherwise.

Response

Thank you for the reminder. We carefully reviewed the reviewer comments and noted that no specific recommendations for additional citations were provided.

Comment 8

Please ensure that you refer to Table 9 in your text as, if accepted, production will need this reference to link the reader to the Table.

Response

Thank you for the reminder. We have revised the manuscript and added the corresponding in-text citation to Table 9 to ensure that the table is properly referenced in the text.

Comment 9

We note that your author list was updated during the revision process. In order to add or remove authors or update the order of the author byline after initial submission, we ask that authors complete an Authorship Change Request form. You may review our full authorship change policy and download the Authorship Change Request form here: https://journals.plos.org/plosone/s/authorship#loc-authorship-changes.

Response

Thank you for the reminder. We have completed the Authorship Change Request form in accordance with the journal’s policy and submitted it along with the revised manuscript.

To reviewer 1

1. Introduction

Comment 1

The theories mentioned will need to be combined to demonstrate how they interact. It is recommended to narrow the theoretical scope by emphasising the most central mechanism.

Response

We sincerely thank the reviewer for this suggestion. We have streamlined the theoretical framework by positioning the Meaning Maintenance Model (MMM) as the primary overarching theory. In the revised Introduction and Discussion, we now explicitly articulate how the MMM integrates social comparison and self-defense mechanisms. Specifically, we clarify the interaction between "meaning violation" (caused by social comparison) and the subsequent paths of defensive compensation (Study 1) and constructive structural realignment (Study 2). This refinement ensures a more focused and cohesive explanation of the core psychological mechanisms at play.

Comment 2

Some key constructs, such as imperfect role models, hostility, and meaning in life, lack operational precision. Provide clear, operational definitions for each construct.

Response

We appreciate this important suggestion. In the revised manuscript, we added explicit operational definitions for the core constructs. Specifically, imperfect role models are defined as successful individuals whose narratives explicitly include experiences of failure or struggle prior to achievement. Hostility is defined as a stable individual difference reflecting a tendency to perceive interpersonal situations as competitive or threatening and to respond with defensive antagonism. Meaning in life is defined as individuals’ perceived sense of purpose, coherence, and significance in their lives. These definitions have been incorporated into the Introduction and Methods sections to clarify the conceptual and empirical scope of each construct.

Comment 3

Strengthen the empirical rationale by explicitly citing inconsistencies or gaps in prior studies.

Response

Thank you for this valuable suggestion. In response, we expanded the literature review to more clearly highlight existing gaps and inconsistencies in prior research. Specifically, we emphasize three unresolved issues: (1) prior studies have primarily focused on the negative consequences of upward social comparison, leaving its potential constructive effects underexplored; (2) empirical research on imperfect role models remains limited compared to the extensive literature on idealized successful figures; and (3) previous work rarely integrates defensive responses and meaning reconstruction within a unified theoretical framework. By addressing these gaps, the revised manuscript clarifies the theoretical and empirical motivation for the present research.

2. Methodology

Comment 4

Sample homogeneity: predominantly female university students limit external validity. It should be discussed in the Discussion Section (Gender Differences) and also included in the Limitations Section.

Response

We sincerely appreciate the reviewer’s suggestion to address the potential influence of uncontrolled variables. In the revised manuscript, we have added a dedicated discussion in the Limitations section to acknowledge these factors. Specifically, we analyzed how variables such as gender distribution (notably the higher proportion of female participants), cultural background, and social desirability bias may have influenced participants’ self-reporting of distress and meaning in life. We have also suggested that future studies could employ more diverse samples and control for social desirability to further validate the generalizability of our findings. This addition provides a more comprehensive and balanced perspective on the scope of our results.

Comment 5

Self-selection bias: participants who attend “failure-sharing sessions” may already favour disclosure. It should be discussed in the Discussion section (Gender Differences) and also included in the limitations section.

Response

We sincerely thank the reviewer for pointing out this critical methodological issue. We agree that participants’ initial willingness to engage with failure-related content may be confounded by their trait-level predisposition toward self-disclosure (self-selection bias). It is also important to clarify that in the present study, participation willingness refers to individuals’ willingness to attend a subsequent experience-sharing session, rather than indicating that they would necessarily disclose their own experiences during the session.

To address this concern, we have implemented the following revisions:

In the Discussion (Section 5.2): We have added an analysis exploring whether "participation willingness" may partially reflect pre-existing disclosure tendencies. We argue that while such a tendency might exist, the significant difference between the prosperity-perception group and the adversity-perception group suggests that the situational interpretation (imperfect role model) still plays a crucial moderating role beyond individual traits.

In the Limitations (Section 5.4.1): We have added a new limitation (the seventh point) specifically addressing this potential self-selection bias. We acknowledge that future research should measure baseline disclosure tendencies or adopt a forced-exposure design to better isolate the causal effect of the experimental manipulation.

Comment 6

Lack of manipulation checks for scenario validity in Study 2.

Response

We sincerely appreciate the reviewer’s suggestion to ensure the internal validity of our experimental manipulation. In the revised manuscript, we have now included a detailed manipulation check in Section 3.2.2. To verify the effectiveness of the scenarios (Failure vs. Success), we performed a Chi-square test between the experimental groups and participants’ subjective perceptions (Adversity vs. Prosperity). The results indicated a highly significant association, χ²(1, N = 536) = 312.87, p < .001, φ = 0.76, with 89.2% of the failure group perceiving the scenario as adversity and 87.5% of the success group perceiving it as prosperity. Furthermore, an additional Chi-square analysis confirmed that the experimental manipulation did not significantly impact participation willingness, χ²(1, N = 536) = 0.14，p = 0.712，φ = -0.016, thereby ruling out participation intent as a confounding variable. These results provide a robust foundation for our subsequent mediation analyses.

Comment 7

The Psychometric properties in brief of the original measures, as well as the translated versions, will be needed to add to each measure. Additionally, some items from each measure are preferred to be included in each measure.

Response

We sincerely appreciate the reviewer’s constructive suggestion. In the revised manuscript (Sections 3.1.2 and 3.2.3), we have comprehensively updated the descriptions for all measurement scales. Specifically, we have added:

Psychometric Properties: We now report the Cronbach’s α coefficients for the original English versions, the previously validated Chinese versions, and the current study’s internal consistency (including sub-dimension reliability where applicable).

Sample Items: Specific example items have been included for each scale to enhance clarity.

Comment 8

Ethical consideration: no debriefing procedures (participants’ support) mentioned after inducing failure scenarios. This needs to be mentioned in the Methodology Section.

Response

We thank the reviewer for this insightful suggestion. In the revised manuscript, we have added a detailed description of the debriefing procedure in Section 3.2.2. Specifically, we clarified the experimental nature of the failure scenarios to the participants and provided a multi-layered support system, including immediate on-site psychological guidance and contact information for the university’s counseling services.

3. Results

Comment 9

Clarify in the response letter how PROCESS was used to assess the CFA.

Response

We are grateful for the opportunity to clarify our statistical approach. We would like to clarify that PROCESS (Hayes, 2022) was utilized exclusively for testing our mediation, serial mediation, and moderated mediation hypotheses (Models 4, 6, and 7), rather than for performing Confirmatory Factor Analysis (CFA). In our data analysis workflow, we focused on path analysis and the estimation of indirect effects using the bias-corrected percentile Bootstrap method. We have carefully audited our manuscript and removed any ambiguous phrasing that might have suggested the use of PROCESS for CFA. Instead, we have explicitly stated that PROCESS was employed to evaluate the proposed theoretical model and the significance of the paths between variables. This clarification ensures that our statistical reporting aligns strictly with the functions of the software used

4. Discussion

Comment 10

Temper causal language (use “may” or “suggests” instead of “reveals” or “proves”).

Response

We sincerely thank the reviewer for this valuable guidance on academic precision. We have carefully audited the entire manuscript, particularly the Discussion section, to refine our phrasing. To address this concern, we have replaced absolute causal terms such as "reveals," "proves," or "determines" with more appropriate, nuanced expressions.

Comment 11

Acknowledge and discuss uncontrolled variables (e.g., gender, culture, social desirability).

Response

We sincerely appreciate the reviewer’s suggestion to address the potential influence of uncontrolled variables. In the revised manuscript, we have added a dedicated discussion in the Limitations section to acknowledge these factors. Specifically, we analyzed how variables such as gender distribution (notably the higher proportion of female participants), cultural background, and social desirability bias may have influenced participants’ self-reporting of distress and meaning in life. We have also suggested that future studies could employ more diverse samples and control for social desirability to further validate the generalizability of our findings. This addition provides a more comprehensive and balanced perspective on the scope of our results.

Comment 12

Distinguish empirical results from theoretical interpretation explicitly.

Response

We thank the reviewer for this insightful feedback on the clarity of our presentation. We have carefully restructured the Discussion section to clearly distinguish between direct e

Attachment Submitted filename: Response to Reviewers.docx

# Editor report: Decision Letter 1

**DOI:** 10.1371/journal.pone.0350109.r003

**Authors:** Atreyee Bhattacharyya

10 May 2026

<p>From defense to reconstruction: The hostility-meaning dual-path model of how observing others’ adversity influences distress disclosure

PONE-D-25-31226R1

Dear Dr. Yuting Wang,

We’re pleased to inform you that your manuscript has been judged scientifically suitable for publication and will be formally accepted for publication once it meets all outstanding technical requirements.

Within one week, you’ll receive an e-mail detailing the required amendments. When these have been addressed, you’ll receive a formal acceptance letter and your manuscript will be scheduled for publication.

An invoice will be generated when your article is formally accepted. Please note, if your institution has a publishing partnership with PLOS and your article meets the relevant criteria, all or part of your publication costs will be covered. Please make sure your user information is up-to-date by logging into Editorial Manager at Editorial Manager® and clicking the ‘Update My Information’ link at the top of the page. For questions related to billing, please contact billing support.

If your institution or institutions have a press office, please notify them about your upcoming paper to help maximize its impact. If they’ll be preparing press materials, please inform our press team as soon as possible -- no later than 48 hours after receiving the formal acceptance. Your manuscript will remain under strict press embargo until 2 pm Eastern Time on the date of publication. For more information, please contact onepress@plos.org.

Kind regards,

Atreyee Bhattacharyya, Ph.D.

Guest Editor

PLOS One

# Editor report: Acceptance letter

**DOI:** 10.1371/journal.pone.0350109.r004

**Authors:** Atreyee Bhattacharyya

PONE-D-25-31226R1

PLOS One

Dear Dr. Wang,

I’m pleased to inform you that your manuscript has been deemed suitable for publication in PLOS One. Congratulations! Your manuscript is now being handed over to our production team.

At this stage, our production department will prepare your paper for publication. This includes ensuring the following:

* All references, tables, and figures are properly cited

* All relevant supporting information is included in the manuscript submission,

* There are no issues that prevent the paper from being properly typeset

You will receive further instructions from the production team, including instructions on how to review your proof when it is ready. Please keep in mind that we are working through a large volume of accepted articles, so please give us a few days to review your paper and let you know the next and final steps.

Lastly, if your institution or institutions have a press office, please let them know about your upcoming paper now to help maximize its impact. If they’ll be preparing press materials, please inform our press team within the next 48 hours. Your manuscript will remain under strict press embargo until 2 pm Eastern Time on the date of publication. For more information, please contact onepress@plos.org.

You will receive an invoice from PLOS for your publication fee after your manuscript has reached the completed accept phase. If you receive an email requesting payment before acceptance or for any other service, this may be a phishing scheme. Learn how to identify phishing emails and protect your accounts at https://explore.plos.org/phishing.

If we can help with anything else, please email us at customercare@plos.org.

Thank you for submitting your work to PLOS ONE and supporting open access.

Kind regards,

PLOS ONE Editorial Office Staff

on behalf of

Dr. Atreyee Bhattacharyya

Guest Editor

PLOS One
